# SUCLG2 Regulates Mitochondrial Dysfunction through Succinylation in Lung Adenocarcinoma

**DOI:** 10.1002/advs.202303535

**Published:** 2023-10-30

**Authors:** Qifan Hu, Jing Xu, Lei Wang, Yi Yuan, Ruiguang Luo, Mingxi Gan, Keru Wang, Tao Zhao, Yawen Wang, Tianyu Han, Jian‐Bin Wang

**Affiliations:** ^1^ Department of Thoracic Surgery The First Affiliated Hospital of Nanchang University Nanchang Jiangxi 330006 China; ^2^ School of Basic Medical Sciences Nanchang University Nanchang Jiangxi 330031 China; ^3^ Jiangxi Institute of Respiratory Disease The First Affiliated Hospital of Nanchang University Nanchang Jiangxi 330006 China; ^4^ School of Huankui Academy Nanchang University Nanchang Jiangxi 330031 China; ^5^ Jiangxi Clinical Research Center for Respiratory Diseases Nanchang Jiangxi 330006 China; ^6^ China‐Japan Friendship Jiangxi Hospital National Regional Center for Respiratory Medicine Nanchang Jiangxi 330200 China

**Keywords:** mitochondrial dysfunction, SIRT5, succinylation, SUCLG2, TRIM21

## Abstract

Mitochondrial dysfunction and abnormal energy metabolism are major features of cancer. However, the mechanisms underlying mitochondrial dysfunction during cancer progression are far from being clarified. Here, it is demonstrated that the expression level of succinyl‐coenzyme A (CoA) synthetase GDP‐forming subunit β (SUCLG2) can affect the overall succinylation of lung adenocarcinoma (LUAD) cells. Succinylome analysis shows that the deletion of SUCLG2 can upregulate the succinylation level of mitochondrial proteins and inhibits the function of key metabolic enzymes by reducing either enzymatic activity or protein stability, thus dampening mitochondrial function in LUAD cells. Interestingly, SUCLG2 itself is also succinylated on Lys93, and this succinylation enhances its protein stability, leading to the upregulation of SUCLG2 and promoting the proliferation and tumorigenesis of LUAD cells. Sirtuin 5 (SIRT5) desuccinylates SUCLG2 on Lys93, followed by tripartite motif‐containing protein 21 (TRIM21)‐mediated ubiquitination through K63‐linkage and degradation in the lysosome. The findings reveal a new role for SUCLG2 in mitochondrial dysfunction and clarify the mechanism of the succinylation‐mediated protein homeostasis of SUCLG2 in LUAD, thus providing a theoretical basis for developing anti‐cancer drugs targeting SUCLG2.

## Graphical abstract

In LUAD, succinylation on Lys93 increases SUCLG2 stability, leading to metabolic reprogramming and tumor progression. SIRT5 desuccinylates SUCLG2 on Lys93, followed by TRIM21‐mediated ubiquitination through K63‐linkage and degradation in the lysosome, this leads to the upregulation of succinyl‐CoA and protein succinylation in the mitochondria and inhibits mitochondrial function. This study discovers a new function for SUCLG2 in tumor progression.

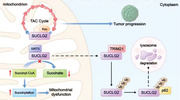

## 1 Introduction

Metabolic reprogramming is one of the 14 hallmarks of cancer.^[^ ^1^ ^]^ As mitochondria form the metabolic centers of cells, their dysfunction is associated with many physiological diseases, including cancer. Succinyl‐coenzyme A ligase (SUCL) is one of the key enzymes in the tricarboxylic acid (TCA) cycle, converting succinyl‐coenzyme A (CoA) to succinate. SUCL has two subunits, the α subunit encoded by SUCLG1 and the β subunit encoded by either the ATP‐forming SUCLA2 or the GTP‐forming SUCLG2. SUCLG2 is expressed in most tissues, especially in tissues with high metabolism and fast protein turnover. Besides its role as a metabolic enzyme in the TCA cycle, SUCLG2 has been shown to maintain mitochondrial DNA (mtDNA) stability by promoting the activity of mitochondrial nucleoside diphosphate kinase (NDPK).^[^ ^2^ ^]^ In cancer cells, SUCLG2 can be regulated at the transcriptional level. In prostate cancer, leukemia inhibitory factor receptor (LIFR) was found to upregulate the mRNA expression of SUCLG2 by activating signal transducer and activator of transcription 3 (STAT3), thus promoting the proliferation of prostate cancer cells and neuroendocrine differentiation (NE).^[^ ^3^ ^]^ The SUCLG2 sequence is highly conserved. However, SUCLG2 is mutated in 4.3% of patients with pheochromocytoma and paraganglioma, and its deletion results in aberrant respiration and elevated the succinate‐to‐fumarate ratio.^[^ ^4^ ^]^ These observations demonstrate that SUCLG2 may play a pivotal role in cancer initiation and progression. However, the function and mechanism of SUCLG2 in lung cancer progression have not been studied.

Post‐translational modifications (PTMs) such as acetylation, succinylation, malonylation, and glutarylation expand the functional diversity of substrate proteins by affecting their expression or activity.^[^ ^5^ ^]^ Succinylation is the process of covalently binding succinyl groups to the lysine residues of substrate proteins to affect substrate protein function.^[^ ^6^ ^]^ Succinylation can change the spatial structure and charged properties of substrate proteins and thus regulates various signaling pathways, including the TCA cycle, the electron transport chain, glycolysis, fatty acid oxidation, and the urea cycle.^[^ ^7^, ^8^, ^9^, ^10^ ^]^ Furthermore, succinylation can promote the growth of various tumors by regulating the succinylation of cancer‐related proteins.^[^ ^11^, ^12^, ^13^ ^]^ Sirtuin 5 (SIRT5), the main desuccinylation enzyme, catalyzes nicotinamide adenine dinucleotide (NAD^+^)‐dependent desuccinylation and localizes primarily in the mitochondrial matrix.^[^ ^14^ ^]^ SIRT5 was reported to be associated with tumor occurrence and development and played different roles in different cancers as either an oncogene or a tumor suppressor.^[^ ^15^, ^16^ ^]^ In lung cancer, SIRT5 can reduce the succinylation level of superoxide dismutase 1 (SOD1), thus reducing reactive oxygen species (ROS) levels in cells by improving SOD1 activity.^[^ ^17^ ^]^ However, the regulatory mechanisms underlying the succinylation of mitochondrial proteins and the key factors involved in this process still need to be further clarified.

In the current study, we found that SUCLG2, but not SUCLA2, was overexpressed in lung adenocarcinoma (LUAD) and closely related to patients’ survival. SUCLG2 knockdown induced mitochondrial dysfunction and significantly inhibited the proliferation of LUAD cells. We found that SUCLG2 knockout increased the succinylation level of mitochondrial proteins and inhibited the function of key metabolic enzymes by reducing either enzymatic activity or protein stability. We also discovered that SUCLG2 was succinylated on Lys93, which enhanced its protein stability in LUAD cells. SIRT5, the desuccinylation enzyme, was found to interact with SUCLG2 and desuccinylate SUCLG2 on Lys93. This led to the lysosome‐mediated degradation of SUCLG2 by the E3 ubiquitin ligase tripartite motif‐containing protein 21 (TRIM21). Thus, our study elucidates the role of SUCLG2 in mitochondrial dysfunction and clarifies the mechanism of the succinylation‐mediated protein homeostasis of SUCLG2 in LUAD, thereby providing a theoretical basis for developing anti‐tumor drugs by targeting SUCLG2.

## 2 Results

### 2.1 SUCLG2 is Essential for the Proliferation of LUAD Cells and Closely Related to the Poor Survival of LUAD Patients

**Figure 1:**
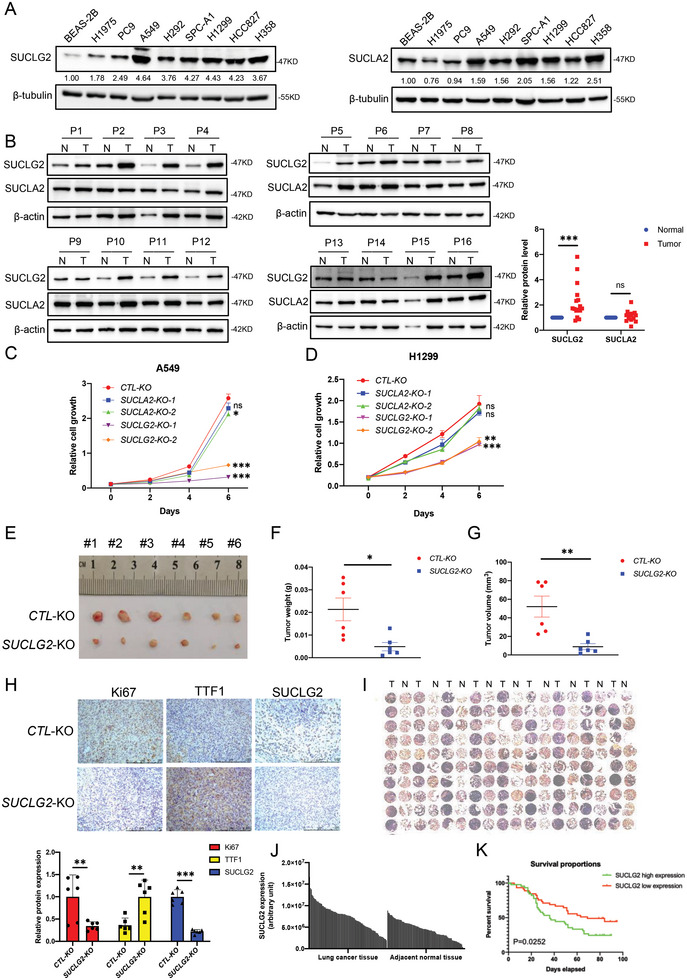
SUCLG2 is essential for the proliferation of LUAD cells and closely related to the poor survival of LUAD patients. A) The protein levels of SUCLG2 and SUCLA2 were detected by western blotting in LUAD cell lines and human bronchial epithelial cell line BEAS‐2B. B) The protein levels of SUCLG2 and SUCLA2 in paired LUAD tissues and adjacent normal tissues were detected by western blotting (left‐hand panels). N: adjacent normal tissue, T: tumor tissue. Protein quantification was performed using ImageJ software (right‐hand panel), and the results represent the average of 16 independent experiments (mean ± SD). ns > 0.05, ***p < 0.001. C and D) A549 cells and H1299 cells with or without SUCLG2 were seeded in 24‐well plates. At the indicated times, the cells were fixed with 4% paraformaldehyde and stained with 1% crystal violet. The dye was extracted with 10% acetic acid, and the relative proliferation was assessed based on the increase in absorbance at 595 nm. The data represent the average of three independent experiments (mean ± SD). ns: *p* > 0.05, *p < 0.05, **p < 0.01, ***p < 0.001. E–G) A549 cells (1 × 10^7^) with CTL‐KO or SUCLG2 depletion (SUCLG2‐KO) were subcutaneously injected into the flanks of nude mice (six mice per group). Twenty‐eight days later, the tumors were dissected out and photographed (E). The weight (F) and volume (G) of the xenograft tumors were measured. The P value was calculated by paired t‐test: *p < 0.05, **p < 0.01. H) Representative IHC images of xenograft tumors with anti‐Ki67 (left), anti‐TTF1 (middle), and SUCLG2 (right) antibodies (upper panels). Quantification of the IHC staining was performed using ImageJ software (bottom panel), and the results represent the average of six independent experiments (mean ± SD). **p < 0.01, ***p < 0.001. I) Microscopic evaluation of the IHC staining of a LUAD tissue microarray with an anti‐SUCLG2 antibody. N: adjacent normal tissue, T: tumor tissue. J) Quantification of the IHC staining shown in Figure 1I. K) Kaplan–Meier survival curve of 90 patients in the LUAD tissue microarray. Patient tissues were divided into two groups based on the average staining density of SUCLG2 in the tumor tissues of the tissue array (high expression: *n* = 45, low expression: *n* = 45; a log‐rank [Mantel–Cox] test was used for the statistical analysis).

GDP‐specific succinyl‐CoA synthetase (SUCLG2) and ATP‐specific succinyl‐CoA synthetase (SUCLA2) are two distinct β subunits of succinyl‐CoA synthetase. To investigate the role of SUCL in LUAD, we first examined the protein expression of SUCLG2 and SUCLA2 in LUAD cells and tissues. We found that the protein expression of SUCLG2 in all LUAD cells was significantly upregulated compared with that in the human bronchial epithelial cell line BEAS‐2B. SUCLA2 was upregulated in some LUAD cells, but others showed downregulation compared with BEAS‐2B, and no consistent trend was seen (**Figure** **1**A). Similar results were obtained when we examined SUCLG2 and SUCLA2 expression in LUAD tissues. Figure 1B shows that the expression of SUCLG2 in LUAD tissues was higher than that in adjacent normal tissues, while SUCLA2 showed no significant changes in most tissue pairs. To investigate the function of SUCLG2 and SUCLA2 in the proliferation of LUAD cells, we depleted SUCLG2 or SUCLA2 in A549 and H1299 cells using CRISPR‐Cas9. Figures S1A and B show that SUCLG2 and SUCLA2 were successfully knocked out in A549 and H1299 cells. Then, we performed cell proliferation assays. The proliferation rates of LUAD cells were markedly attenuated when SUCLG2, but not SUCLA2, was knocked out (Figure 1C,D). Thus, these results demonstrated that SUCLG2, rather than SUCLA2, plays a major role in LUAD.

To further detect the effects of SUCLG2 on the proliferation of LUAD cells, we knocked down SUCLG2 in LUAD cell lines (H292, H23, HCC827, and SPC‐A1) and BEAS‐2B cells using specific siRNAs, then performed cell growth assays. Knocking down SUCLG2 significantly inhibited the proliferation of LUAD cells but not BEAS‐2B cells (Figure S1C—G, Supporting Information). Colony formation assays also showed that knocking down SUCLG2 significantly attenuated the colony‐forming ability of LUAD cells, indicating that SUCLG2 is essential for the proliferation of LUAD cells (Figure S1H—J, Supporting Information). Furthermore, we performed a xenograft assay to determine the tumorigenesis of A549 wild‐type (WT) and SUCLG2 knockout (SUCLG2‐KO) cells. A549 SUCLG2‐KO showed a decrease in tumor weight and volume compared with A549‐WT cells (Figure 1E–G). To confirm these results, we performed immunohistochemistry (IHC) analysis of the tumor samples of A549‐WT and A549‐SUCLG2‐KO using anti‐Ki67, anti‐TTF1, and anti‐SUCLG2 antibodies. Ki67 is widely used as a proliferation marker in pathological assessments, while TTF1 has been demonstrated to be frequently suppressed in high‐grade LUAD. Our results showed that the staining of Ki67 and SUCLG2 was markedly higher in the A549‐WT group than in the A549‐SUCLG2‐KO group. However, the staining of TTF1 was lower in the A549‐WT group than in the A549‐SUCLG2‐KO group (Figure 1H). To further determine whether the high expression of SUCLG2 in LUAD has clinical relevance, we examined the protein expression of SUCLG2 in a LUAD tissue array by IHC using the anti‐SUCLG2 antibody. The tissue array included lung cancer tissues and adjacent normal tissues from 90 LUAD patients. The results demonstrated that tumor tissues expressed significantly higher levels of SUCLG2 compared to the adjacent normal tissues (Figure 1I); this result was further validated by quantification of the staining (Figure 1J). Statistical analysis of the staining quantification results of the cancer tissues divided the samples into two groups depending upon the SUCLG2 level. Patient survival was correlated with the SUCLG2 level. As shown in Figure 1K, patients with low levels of SUCLG2 exhibited better survival than those with high levels of SUCLG2 (P = 0.0252). These results indicated that SUCLG2 affects the proliferation and tumorigenesis of LUAD cells and is closely related to LUAD progression and patient survival.

### 2.2 SUCLG2 Deficiency Leads to Mitochondrial Dysfunction

**Figure 2:**
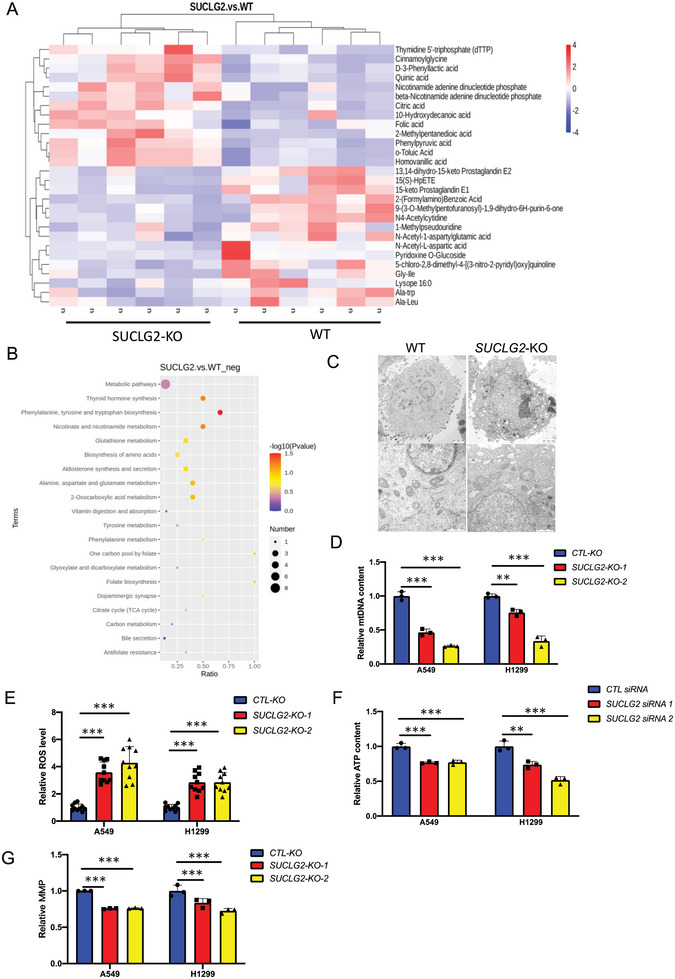
SUCLG2 knockout induces mitochondrial dysfunction by affecting the metabolism of LUAD cells. A and B) A549‐SUCLG2‐KO and A549‐WT cells were used for untargeted negative ion mode metabolomics analysis (A). The negative ion mode metabolomics relative pathway analysis is shown (B). *n* = 6 per group. C) Mitochondrial morphology was observed by TEM. Scale bars: 2 µm (upper panels) and 1 µm (bottom panels). D) qPCR analysis of relative mtDNA content. Data represent the average of three independent experiments (mean ± SD). **p < 0.01, ***p < 0.001. E) ROS generation was assessed with 5 µM H2DCFDA added to A549 and H1299 cells or SUCLG2 knockout stable cell lines, followed by confocal microscopy. Data represent the average of three independent experiments (mean ± SD). ***p < 0.001. F) A549 and H1299 cells were transfected with individual siRNAs targeting SUCLG2. The intracellular ATP concentration was determined based on luminescence values and normalized to the protein content in each sample. Data represent the average of three independent experiments (mean ± SD). **p < 0.01, ***p < 0.001. G) The MMP of A549 and H1299 cells and SUCLG2 knockout stable cell lines was determined by adding JC‐1 and measuring immunofluorescence based on the absorbance at 490 nm/530 nm. Data represent the average of three independent experiments (mean ± SD). ***p < 0.001.

Mitochondria are the central organelles of metabolic activities, and SUCLG2 is a key enzyme in the TCA cycle. Thus, we speculated that SUCLG2 might affect tumor proliferation by regulating mitochondrial metabolism. We performed untargeted metabolomics in A549‐WT and A549‐SUCLG2‐KO cells (Table S1, Supporting Information) and found that 15 metabolites were upregulated, 64 metabolites were downregulated in A549‐SUCLG2‐KO cells compared with A549‐WT cells (**Figure** **2**A; Figure S2A, Supporting Information). We analyzed the metabolites using KEGG pathway analysis and found that mitochondria‐related metabolic pathways like the TCA cycle and glutathione metabolism were significantly changed (Figure 2B; Figure S2B, Supporting Information). To detect the effect of SUCLG2 on mitochondrial function, we performed transmission electronic microscopy (TEM). A549 cells were found to contain mitochondria with tubular shapes and clear cristae, while A549‐SUCLG2‐KO cells exhibited swollen mitochondria with fractured cristae (Figure 2C). The mtDNA copy number has been demonstrated to be increased in many types of cancer, including lung cancer.^[^ ^18^, ^19^, ^20^, ^21^, ^22^ ^]^ We found that SUCLG2 knockout in A549 and H1299 cells decreased the mtDNA level (Figure 2D). In addition, the cellular ROS level was increased in SUCLG2‐KO cells compared to that in control cells (Figure 2E). The ATP content in A549 and H1299 cells was decreased after SUCLG2 knockdown (Figure 2F). Thereafter, we measured the mitochondrial membrane potential (MMP) in A549 and H1299 cells with SUCLG2 knockout. SUCLG2 knockout cells had a significantly lower MMP compared with control cells (Figure 2G). From these results, we concluded that the maintenance of mitochondrial function in LUAD cells depends on the expression of SUCLG2.

### 2.3 SUCLG2 Knockout Induces a Global Increase in Protein Succinylation

**Figure 3:**
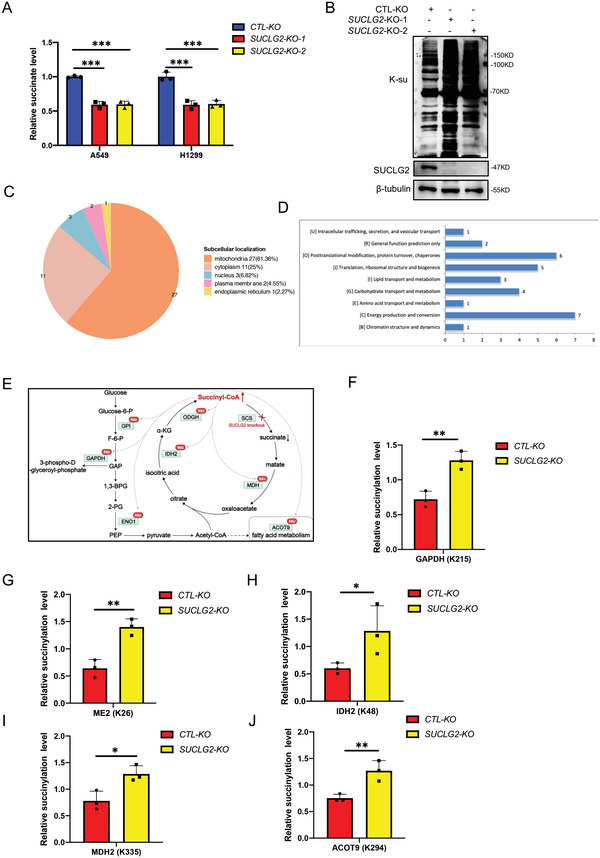
SUCLG2 knockout induces a global increase in protein succinylation. A) Succinate levels in A549 and H1299 cells and SUCLG2 knockout stable cell lines. Data represent the average of three independent experiments (mean ± SD). ***p < 0.001. B) The succinylation of proteins was detected in A549‐SUCLG2‐KO and A549‐WT cells using western blotting. C and D) The succinylation of proteins in A549‐SUCLG2‐KO and A549‐WT cells was analyzed with a 4D mass spectrometer. Intracellular distribution of succinylated proteins (C). Biological process analysis showing that upregulated succinylated proteins were significantly enriched in processes related to mitochondrial dysfunction (D). *n* = 3 per group. E) Schematic depicting the key enzymes related to mitochondrial function by knock outing SUCLG2. F–J) The succinylation levels of GAPDH, ME2, IDH2, MDH2, and ACOT9 in A549‐SUCLG2‐KO and A549‐WT cells measured by mass spectrometry. Data represent the average of three independent experiments (mean ± SD). *p < 0.05, **p < 0.01.

SUCLG2 is the main hydrolase of succinyl‐CoA, which is the main succinyl‐modified substrate for protein succinylation. Succinylation is considered to be a nonenzymatic reaction regulated by the concentration of the succinyl‐CoA donor.^[^ ^23^ ^]^ Therefore, we speculated that SUCLG2 might regulate mitochondrial function and metabolic reprogramming through succinylation. Because succinyl‐CoA is subject to oxidative hydrolysis, it is hard to detect. Thus, we detected the content of succinic acid, the product of succinyl‐CoA catalyzed by SUCLG2, in A549 and H1299 cells with SUCLG2 knockout. **Figure** **3**A shows that SUCLG2 knockout significantly reduced the content of succinic acid. We next examined the effect of SUCLG2 on the succinylation level of intracellular proteins. The results showed that SUCLG2 knockout increased the succinylation level of intracellular proteins (Figure 3B). Then, we performed succinyl 4D mass spectrometry analysis to further study the succinylation of proteins regulated by SUCLG2. The results revealed 686 succinyl‐lysine (K‐su) sites in 285 proteins and 61 differentially expressed succinylated sites in 44 proteins in cells with SUCLG2 knockout. We found that most of the succinylation identified in A549‐SUCLG2‐KO cells was upregulated, with 61% distributed in the mitochondria, compared with that in A549‐WT cells (Figure 3C). The GO analysis showed that mitochondria‐related metabolic pathways, like energy production and conversion, were significantly changed (Figure 3D). The succinylation of key enzymes like glyceraldehyde‐3‐phosphate dehydrogenase (GAPDH), NAD‐dependent malic enzyme (ME2), isocitrate dehydrogenase (IDH2), malate dehydrogenase (MDH2), and acyl‐coenzyme A thioesterase 9 (ACOT9), which was related to mitochondrial function, was significantly upregulated on specific sites (Figure 3E–J). These results suggested that SUCLG2 knockout promotes the succinylation of proteins associated with mitochondrial function.

### 2.4 SUCLG2 Affects Protein Stability or Enzymatic Activity by Regulating Succinylation

**Figure 4:**
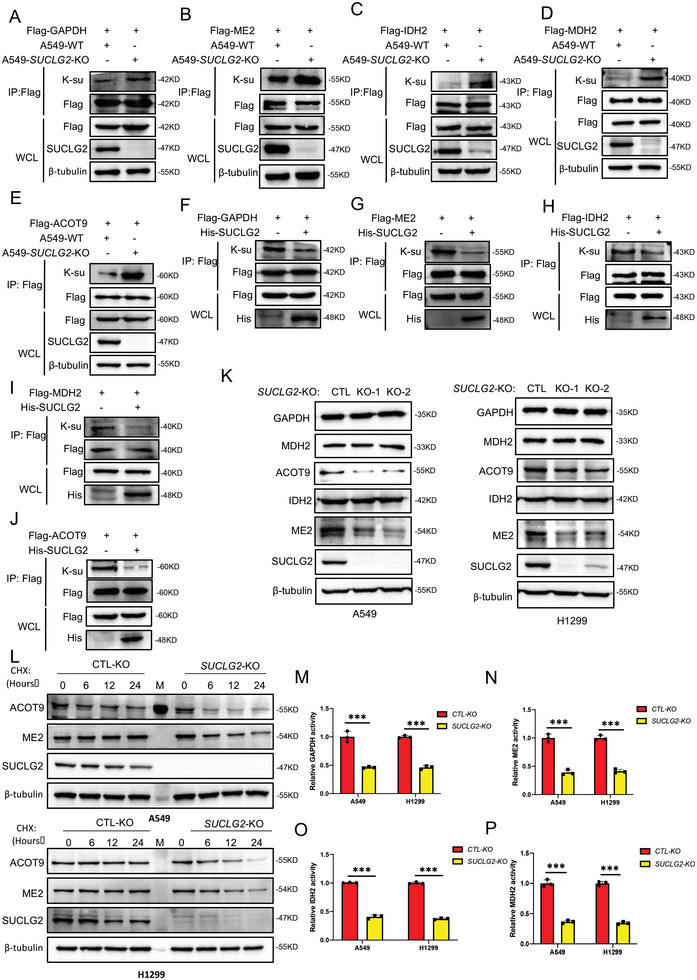
SUCLG2 affects protein stability or enzymatic activity by regulating succinylation. A–E) Western blotting was used to detect the succinylation of the GAPDH, ME2, IDH2, MDH2, and ACOT9 proteins in A549‐WT and A549‐SUCLG2‐KO cells. WCL: whole cell lysate. F–J) Western blotting was used to detect the succinylation of the GAPDH, ME2, IDH2, MDH2, and ACOT9 proteins in cells with or without SUCLG2 overexpression. K) The expression levels of GAPDH, ME2, IDH2, MDH2, and ACOT9 proteins in wild‐type or SUCLG2 knockout stable cell lines were detected by western blotting. L) The cells were treated with 20 µg mL^−1^ CHX and collected at the indicated time. The degradation rate of the ME2 and ACOT9 proteins was detected by western blotting. M–P) The activity of GAPDH, ME2, IDH2, and MDH2 was detected using a specific enzyme activity detection kit. Data represent the average of three independent experiments (mean ± SD). ***p < 0.001.

We next examined the effect of SUCLG2 on the function of key enzymes like GAPDH, ME2, IDH2, MDH2, and ACOT9. We first checked the effects of SUCLG2 on the succinylation of these proteins to verify the succinyl 4D mass spectrometry analysis results. The results showed that SUCLG2 knockout increased the succinylation of GAPDH, ME2, IDH2, MDH2, and ACOT9 (**Figure** **4**A–E), while overexpressing SUCLG2 in A549 cells decreased the succinylation of these proteins (Figure 4F–J). These results confirmed that SUCLG2 regulates the succinylation of mitochondria‐related proteins. We next examined how SUCLG2 affects the function of these metabolic enzymes. First, we checked the expression of GAPDH, ME2, IDH2, MDH2, and ACOT9 in A549 and H1299 cells with SUCLG2 knockout using western blotting. Figure 4K shows that SUCLG2 knockout decreased the protein expression of ME2 and ACOT9, while the expression of GAPDH, IDH2, and MDH2 was not affected. To determine how SUCLG2 regulates the expression of ME2 and ACOT9, we measured the stability of ME2 and ACOT9 in A549‐WT and A549‐SUCLG2‐KO cells. The results showed that ME2 and ACOT9 proteins had faster degradation rates in A549‐SUCLG2‐KO than in A549‐WT cells (Figure 4L). To further confirm that the protein stability was regulated by succinylation, we made point mutations at the succinylation sites of ACOT9 (K157R, K294R) and ME2 (K26R, K94R), which were identified from the succinyl 4D mass spectrometry results. Then, we performed the IP‐WB assay of both WT and mutant ACOT9 (K157R, K294R) and ME2 (K26R, K94R) to identify the succinylation sites of ACOT9 and ME2 (Figure S3A and B, Supporting Information). The protein degradation rates were examined. The degradation of ACOT9^K157R^ was much slower than that of ACOT9^WT^ and ACOT9^K294R^ when cells were treated with CHX (Figure S3C, Supporting Information). While the degradation of ME2^K26R^ was slower than that of ME^WT^ and ME^K94R^ (Figure S3D, Supporting Information). In addition, we found that ACOT9^K157R^ had similar degradation rates in A549‐SUCLG2‐KO and in A549‐WT cells (Figure S3E, Supporting Information), ACOT9 ^K294R^ had faster degradation rates in A549‐SUCLG2‐KO than in A549‐WT cells (Figure S3F, Supporting Information). The degradation rates of ME2^K26R^ did not changed in A549‐SUCLG2‐KO and in A549‐WT cells (Figure S3G, Supporting Information). While ME2 ^K94R^ had faster degradation rates in A549‐SUCLG2‐KO than in A549‐WT cells (Figure S3H, Supporting Information). These results demonstrated that succinylation modulated the protein degradation of ME2 and ACOT9, and K157 was the main succinylation site for ACOT9, and K26 was the main succinylation site for ME2. As the expression of GAPDH, IDH2, and MDH2 was not affected by SUCLG2 knockout, we next tested the enzymatic activity of these enzymes. Knocking out SUCLG2 decreased GAPDH, ME2, IDH2, and MDH2 activity in A549 and H1299 cells (Figure 4M–P). These results indicate that SUCLG2 knockout inhibits the function of key metabolic enzymes associated with mitochondrial function by reducing either enzymatic activity or protein stability.

### 2.5 TRIM21 Induces SUCLG2 Degradation Through the K63‐Linkage Ubiquitination‐Mediated Lysosomal Pathway

**Figure 5:**
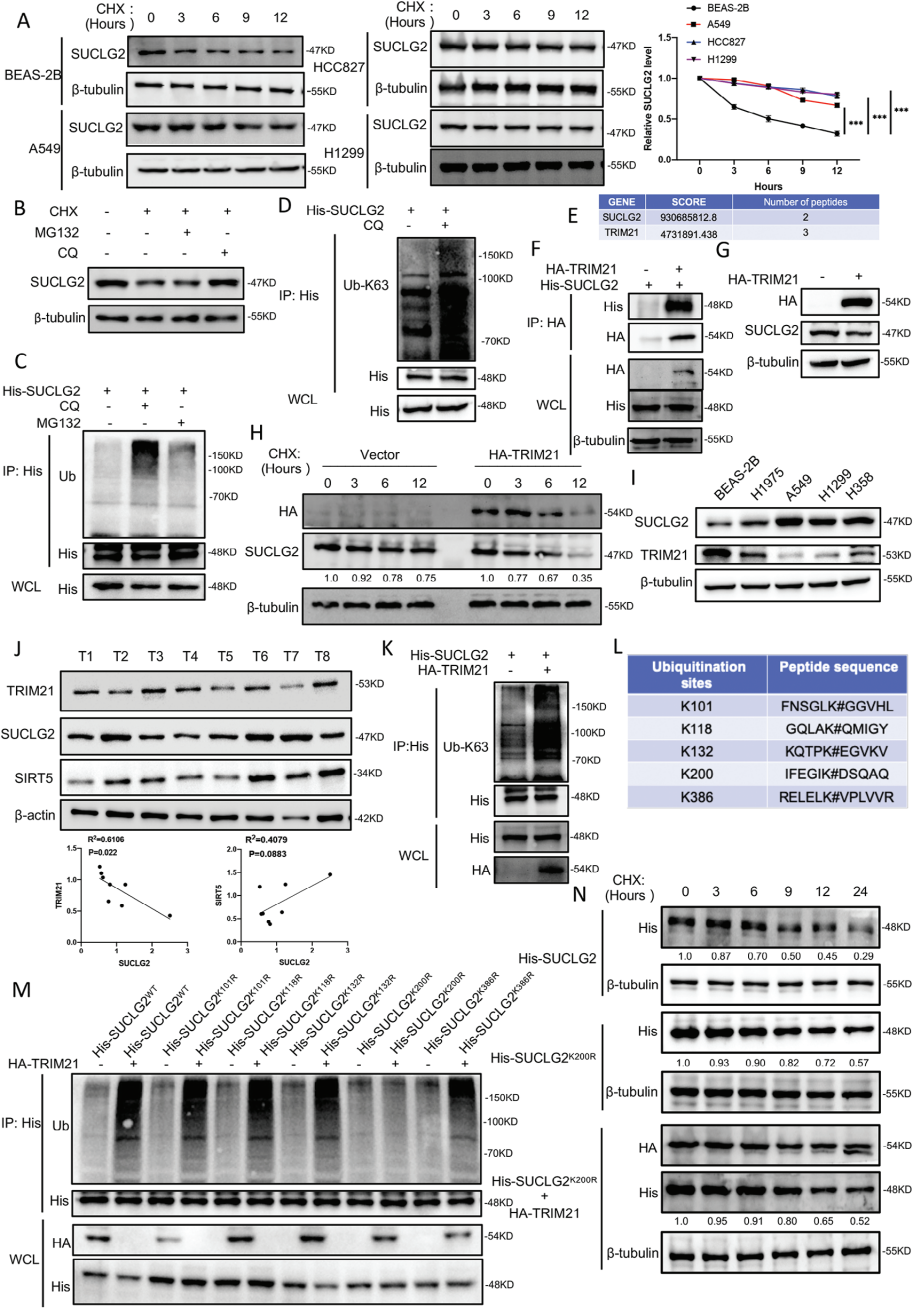
TRIM21 induces SUCLG2 degradation through the K63‐linkage ubiquitin lysosomal pathway. A) BEAS‐2B, A549, HCC827, and H1299 cells were treated with 20 µg mL^−1^ CHX and collected at the indicated time. The degradation rate of SUCLG2 was tested by western blotting (left‐hand panels). Relative SUCLG2 expression was analyzed using ImageJ (right‐hand panel). Data represent the average of three independent experiments (mean ± SD). ***p < 0.001. B) CHX (20 µg mL^−1^) was added to A549 cells for 24 h, and CQ (20 µm) or MG132 (20 µm) was added at the same time. The expression of SUCLG2 was detected using western blotting. C) pcDNA3.1‐His‐SUCLG2 was overexpressed in A549 cells treated with MG132 (20 µm) or CQ (20 µm) for 24 h. Co‐IP and western blotting were used to detect the ubiquitination level of SUCLG2. D) The indicated plasmids were transfected into A549 cells treated with CQ (20 µm) for 24 h. Co‐IP was used to detect the K63‐linkage ubiquitination level of SUCLG2. E) The E3 ligase for SUCLG2 identified by mass spectrometry is shown. F) The interaction between SUCLG2 and TRIM21 was detected using co‐IP in A549 cells transfected with pcDNA3.1‐His‐SUCLG2 and pCMV‐HA‐TRIM21. G) A549 cells were transfected with pCMV‐HA‐TRIM21, and western blotting was used to detect the expression of the indicated proteins. H) A549 cells transfected with or without pCMV‐HA‐TRIM21 were treated with CHX (20 µg mL^−1^) for the indicated times. The protein stability of SUCLG2 was detected by western blotting. I) The protein levels of SUCLG2 and TRIM21 were detected by western blotting in LUAD cell lines and human bronchial epithelial cell line BEAS‐2B. J) The protein levels of SUCLG2, TRIM21, and SIRT5 in 8 LUAD tissues were detected by western blotting (upper panels). T: tumor tissue. Protein correlation analysis was performed using ImageJ software and Prism 8 (bottom panels). K) The indicated plasmids were transfected into A549 cells. Co‐IP and western blotting were used to detect the K63 ubiquitination level of SUCLG2. L) The ubiquitination sites of SUCLG2 predicted by PhosphoSitePlus. M) A549 cells were transfected with the indicated plasmids, and the ubiquitination of SUCLG2 was detected by co‐IP and western blotting. N) A549 cells were transfected with the indicated plasmids, then treated with CHX (20 µg mL^−1^) for 0, 3, 6, 9, 12, and 24 h. The degradation rate of SUCLG2 was detected by western blotting.

As SUCLG2 depletion‐mediated mitochondrial dysfunction contributed to LUAD inhibition, we hypothesized that the downregulation of SUCLG2 might be a potentially feasible strategy to block tumor growth. Thus, clarifying the mechanism of SUCLG2 overexpression in LUAD was important in the following study. We first checked the mRNA levels of SUCLG2 in LUAD cells and BEAS‐2B cells. We found that the mRNA levels of *SUCLG2* were upregulated in some LUAD cells and downregulated in others compared with BEAS‐2B (Figure S4A, Supporting Information). We next analyzed *SUCLG2* TPM (Transcripts Per Kilobase of exon model per Million mapped reads) expression in LUAD using the GEPIA platform and found that *SUCLG2* TPM expression did not differ significantly between tumor and normal tissues (Figure S4B, Supporting Information). This indicated that the upregulation of the SUCLG2 protein in LUAD might not be due to the enhanced transcription of the *SUCLG2* gene. Then, we performed cycloheximide (CHX)‐chase assays to detect the stability of the SUCLG2 protein in LUAD cell lines (A549, HCC827, and H1299) and BEAS‐2B cells. The results showed that the protein degradation of SUCLG2 was markedly accelerated in BEAS‐2B cells compared with that in A549, HCC827, and H1299 cells (**Figure** **5**A). These results suggested that the high expression of SUCLG2 in LUAD was due to enhanced protein stability. Thus, we examined the degradation pathway of SUCLG2. We found that the protein level of SUCLG2 was markedly decreased when A549 cells were treated with CHX for 24 h, and this effect could be recovered by adding the lysosomal inhibitor chloroquine (CQ) but not the proteasomal inhibitor MG132 (Figure 5B). CQ treatment also increased the protein level of SUCLG2 in BEAS‐2B cells (Figure S5A, Supporting Information). In addition, we examined the ubiquitination level of SUCLG2 by adding MG132 or CQ and found that the ubiquitination of SUCLG2 was significantly increased when CQ, but not MG132, was added (Figure 5C). We then tested the ubiquitination types and found that the K63‐linkage ubiquitination of SUCLG2 was up‐regulated in A549 cells treated with CQ (Figure 5D), but the K48‐linkage ubiquitination of SUCLG2 did not show significant changes (Figure S5B, Supporting Information). These results demonstrate that the SUCLG2 protein is degraded by the K63‐linkage ubiquitination‐mediated lysosomal pathway.

To identify the E3 ligase for SUCLG2, we performed mass spectrometry analysis and identified TRIM21 as a putative SUCLG2‐interacting protein (Figure 5E). We verified the interaction between SUCLG2 and TRIM21 in A549 cells using immunoprecipitation (Figure 5F). To determine whether TRIM21 regulated the transcription of SUCLG2, we tested the mRNA expression of *SUCLG2*. Neither overexpression nor knockdown of TRIM21 affected *SUCLG2* mRNA expression (Figures S5C and D). Then, we examined the protein level of SUCLG2 when overexpressing or knocking down TRIM21. The results showed that overexpressing TRIM21 decreased SUCLG2 protein expression in A549 cells (Figure 5G), while TRIM21 knockdown increased SUCLG2 protein expression in A549 and BEAS‐2B cells (Figure S5E and F, Supporting Information). Moreover, overexpressing TRIM21 accelerated the degradation rate of SUCLG2 after CHX treatment (Figure 5H). In contrast, the degradation rate of SUCLG2 was slowed down in A549 and BEAS‐2B cells with TRIM21 knockdown (Figure S5G and H, Supporting Information). Meanwhile, we found that the protein expressions of TRIM21 and SUCLG2 were negatively correlated in LUAD cells and BEAS‐2B cells (Figure 5I). Similar trend was also observed in LUAD tissues. The protein expression of TRIM21 showed a negative correlation with SUCLG2, SIRT5 and SUCLG2 no consistent trend was seen (Figure 5J). We further explored the types of ubiquitin chain linkage of SUCLG2 mediated by TRIM21. Overexpressing TRIM21 increased the K63‐linkage ubiquitination of SUCLG2 (Figure 5K) but not the K48‐linkage (Figure S5I, Supporting Information). However, knocking down TRIM21 significantly decreased the ubiquitination and K63‐linkage ubiquitination level of SUCLG2 (Figure S5J and K, Supporting Information). We next determined if TRIM21 degraded SUCLG2 through the proteasomal pathway or the lysosomal pathway. We found that CQ treatment, but not MG132 treatment, enhanced the ubiquitination of SUCLG2 when TRIM21 was overexpressed (Figure S5L and M, Supporting Information), indicating that TRIM21 ubiquitinated and degraded SUCLG2 via the lysosomal pathway. Altogether, these data supported the model that TRIM21 ubiquitinates SUCLG2 through K63‐linkage, followed by lysosomal degradation.

Sequestosome 1 (p62) plays an important role in the ubiquitin‐lysosome degradation pathway. p62 can deliver the target protein to the lysosome by direct binding.^[^ ^24^ ^]^ We found that p62 and SUCLG2 showed co‐localization in A549 cells as tested by immunofluorescence (Figure S6A, Supporting Information). Next, we verified the interaction between SUCLG2 and p62 through co‐IP. The results showed that SUCLG2 and p62 interacted with each other (Figure S6B and C, Supporting Information). Then, we examined the protein level of SUCLG2 when overexpressing p62. Figures S6D and E show that overexpressing p62 decreased SUCLG2 protein expression in A549 and H1299 cells. Next, we used ubiquitin mutants (K48‐ubiquitin: all the lysine residues were mutated within ubiquitin except for lysine 48; K63‐ubiquitin: all the lysine residues were mutated within ubiquitin except for lysine 63) to explore the binding of p62 to SUCLG2. We found that overexpressing K63‐ubiquitin in A549 could increase the binding of SUCLG2 to p62 (Figure S6F, Supporting Information). Using co‐IP assays, we also revealed that overexpressing TRIM21 promoted the binding of SUCLG2 to p62 in A549 cells (Figures S6G, Supporting Information). These results demonstrated that p62 plays an important role in the process of SUCLG2 degradation.

We next identified the ubiquitination sites of SUCLG2 regulated by TRIM21. Using PhosphoSitePlus, we identified K101, K118, K132, K200, and K386 as putative ubiquitination sites for SUCLG2 (Figure 5L). Then, we constructed SUCLG2 plasmids containing the indicated mutations (K101R, K118R, K132R, K200R, and K386R). We found that only the ubiquitination of SUCLG2^K200R^ did not change when TRIM21 was overexpressed (Figure 5M). We next measured the stability of wild‐type SUCLG2 (SUCLG2^WT^) and the SUCLG2^K200R^ mutant. The degradation of SUCLG2^K200R^ was much slower than that of SUCLG2^WT^ when cells were treated with CHX. Moreover, the overexpression of TRIM21 could not increase the degradation rate of SUCLG2^K200R^ (Figure 5N). These data suggested that K200 of SUCLG2 is the major ubiquitination site for TRIM21.

### 2.6 The K93‐Succinylation of SUCLG2 Affects the TRIM21‐Mediated Degradation of SUCLG2

**Figure 6:**
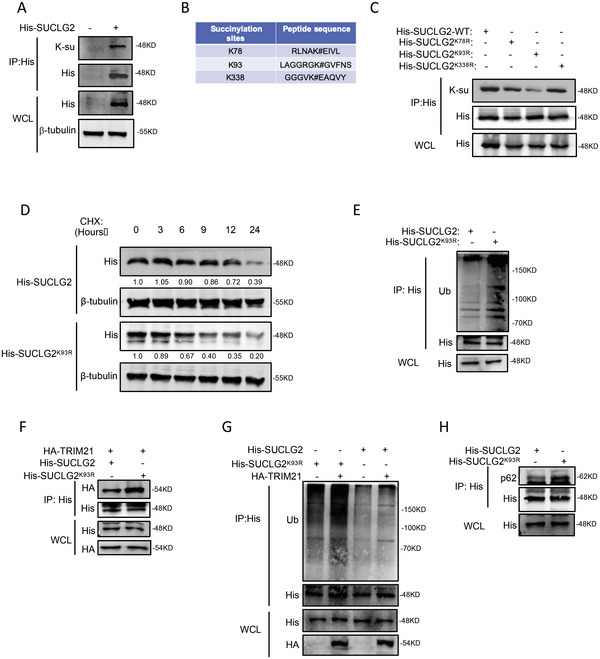
The K93‐succinylation of SUCLG2 affects the TRIM21‐mediated degradation of SUCLG2. A) pcDNA‐3.1‐His‐SUCLG2 was transfected into A549 cells, and co‐IP and western blotting were used to detect the succinylation level of SUCLG2. B) The succinylation sites of SUCLG2 predicted by PhosphoSitePlus. C) A549 cells overexpressing the SUCLG2‐WT or SUCLG2 lysine residue‐mutated plasmids were prepared, and co‐IP and western blotting were used to detect the succinylation of protein. D) A549 cells were transfected with the pcDNA‐His‐SUCLG2 or pcDNA‐His‐SUCLG2^K93R^ plasmids. The protein degradation rate of SUCLG2 was detected by western blotting. E) Differences in ubiquitination levels between SUCLG2‐WT and SUCLG2^K93R^ were assessed. Co‐IP and western blotting were used to detect the ubiquitination level of SUCLG2. F) A549 cells were transfected with the indicated plasmids, and co‐IP and western blotting were used to detect the interaction between SUCLG2 and TRIM21. G) A549 cells were transfected with the indicated plasmids. The ubiquitination of the SUCLG2 protein was detected by co‐IP and western blotting. H) A549 cells were transfected with pcDNA‐His‐SUCLG2 or pcDNA‐His‐SUCLG2^K93R^ plasmids, and co‐IP and western blotting were used to detect the interaction between SUCLG2 and p62.

SUCLG2 is localized to the mitochondrial matrix and is crucial for converting succinyl‐CoA to succinate. The succinyl 4D mass spectrometry analysis results demonstrated that SUCLG2 could also be succinylated (Table S2, Supporting Information). Therefore, we speculated that succinylation affects the function of SUCLG2. First, we demonstrated that the SUCLG2 protein could be succinylated using co‐IP (**Figure** **6**A). Using PhosphoSitePlus and our mass spectrometry results, we identified K78, K93, and K338 as putative succinylation sites for SUCLG2 (Figure 6B). To identify the major succinylation sites for SUCLG2, we constructed SUCLG2 plasmids containing the indicated mutations (K78R, K93R, and K338R) and found that only the succinylation of SUCLG2^K93R^ decreased compared with SUCLG2^WT^ (Figure 6C). In addition, the succinyl 4D mass spectrometry analysis demonstrated that K93 was a succinylation site for SUCLG2 (Table S2, Supporting Information). We next measured the stability of SUCLG2^WT^ and SUCLG2^K93R^. The SUCLG2^K93R^ protein level decreased faster than that of SUCLG2^WT^ when cells were treated with CHX (Figure 6D). These results indicate that K93 of SUCLG2 is the major succinylation site for SUCLG2, and its mutation reduces protein stability in LUAD cells.

We next explored whether SUCLG2 succinylation regulated its ubiquitination. We performed a co‐IP assay and found that the ubiquitination of SUCLG2^K93R^ was stronger compared with that of SUCLG2^WT^ (Figure 6E). This result suggested that the SUCLG2^K93R^ mutant affected the ubiquitination of SUCLG2. We also found that TRIM21 was more inclined to bind SUCLG2^K93R^ and increased its ubiquitination (Figure 6F and G). Furthermore, SUCLG2^K93R^ bound more p62 than SUCLG2^WT^ (Figure 6H). These results confirmed that SUCLG2^K93R^ facilitates its degradation by increasing TRIM21‐mediated ubiquitination and lysosomal degradation.

### 2.7 SIRT5 is the Desuccinylase for SUCLG2 and Affects its Protein Stability

**Figure 7:**
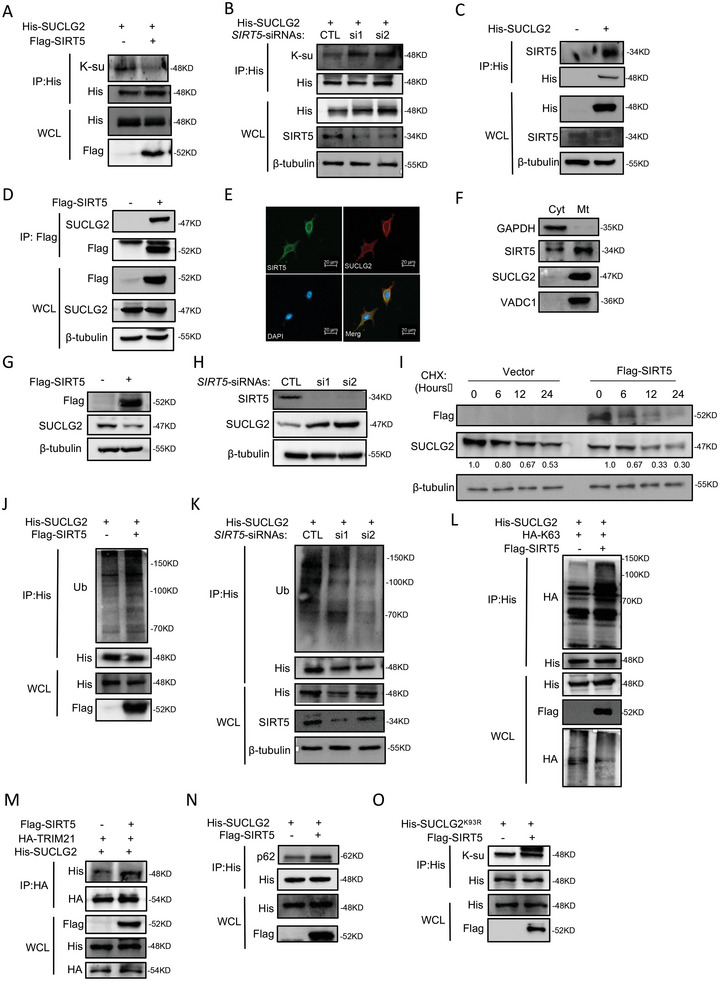
SIRT5 is the desuccinylase for SUCLG2 and affects the degradation of the SUCLG2 protein. A and B) In A549 cells, SIRT5 was overexpressed (A) or knocked down (B) and the succinylation of SUCLG2 was detected by co‐IP and western blotting. C and D) In A549 cells, SUCLG2 (C) or SIRT5 (D) was overexpressed, and the interaction between SIRT5 and SUCLG2 was assessed using co‐IP and western blotting. E and F) The co‐localization of SUCLG2 and SIRT5 was detected by immunofluorescence (E) and mitochondrial isolation, followed by western blotting (F). G and H) In A549 cells, SIRT5 was overexpressed (G) or knocked down (H) and the SUCLG2 protein was detected by western blotting. I) SIRT5 was overexpressed in A549 cells. The degradation rate of the SUCLG2 protein was detected by western blotting. J and K) The indicated plasmids or SIRT5 siRNAs were transfected into A549 cells. Co‐IP and western blotting were used to detect the ubiquitination level of SUCLG2. L) The indicated plasmids were transfected into A549 cells. Co‐IP and western blotting were used to detect the ubiquitination level of SUCLG2. M) Co‐IP was performed in A549 cells transfected with the indicated plasmids before subjecting them to western blotting. N) Co‐IP was performed in A549 cells transfected with SUCLG2 and SIRT5 before subjecting them to western blotting with an anti‐p62 antibody. O) Co‐IP was performed in A549 cells transfected with SUCLG2^K93R^ and SIRT5 before subjecting them to western blotting with a succinylation antibody.

Desuccinylation is mainly regulated by the NAD^+^‐dependent SIRT family. We sought to identify which SIRT was involved in SUCLG2 desuccinylation. Thus far, SIRT5 and SIRT7 have been reported as the desuccinylases in mitochondria. We examined whether SIRT5 and SIRT7 could desuccinylate SUCLG2 and affect its function. In cells overexpressing SIRT5, the succinylated SUCLG2 level was significantly lower than in cells containing the empty vector (**Figure** **7**A). Figure 7B shows that knocking down SIRT5 increased the succinylation of SUCLG2. However, SIRT7 could not affect the succinylation of SUCLG2 (Figure S7A, Supporting Information). Next, we verified the interaction between SUCLG2 and SIRT5 through co‐IP. The results showed that SUCLG2 and SIRT5 interacted with each other (Figure 7C,D; Figure S7B and C, Supporting Information). To further validate their interaction, we performed immuno‐fluorescence and mitochondria isolation assays. Our results showed that SIRT5 co‐located with SUCLG2 in the mitochondria (Figures 7E and F), indicating that SIRT5 interacts with SUCLG2.

We next explored the relationship between SIRT5 and SUCLG2. First, SIRT5 was overexpressed or knocked down, and SUCLG2 expression were detected. SIRT5 knockdown led to an increase in SUCLG2 protein levels, while SIRT5 overexpression decreased SUCLG2 expression (Figure 7G,H; Figure S7D and E, Supporting Information). However, the mRNA level of SUCLG2 was not affected by SIRT5 (Figures S7F and G), suggesting that SIRT5 might affect the protein stability of SUCLG2. We also examined the effect of SIRT5 on SUCLG2 protein homeostasis. Using CHX to block the protein synthesis of SUCLG2, we found that overexpressing SIRT5 accelerated the degradation of SUCLG2 (Figure 7I). Next, we found that overexpressing SIRT5 significantly increased the ubiquitination level of SUCLG2 (Figure 7J), and knocking down SIRT5 reduced the ubiquitination of SUCLG2 (Figure 7K). We further detected the types of ubiquitin chains in SUCLG2 induced by SIRT5. Overexpressing SIRT5 increased the K63‐linkage ubiquitination of SUCLG2 (Figure 7L) but not K48‐linkage ubiquitination (Figure S7H, Supporting Information). Using co‐IP assays, we also revealed that overexpressing SIRT5 promoted the binding of SUCLG2 to TRIM21 and p62 in A549 cells (Figure 7M,N). These results suggested that SIRT5 ubiquitinates SUCLG2 through K63‐linkage, followed by degradation via the lysosomal pathway.

Next, we explored the relationship between SIRT5 and SUCLG2^K93R^. We found that SIRT5 did not affect the succinylation of SUCLG2^K93R^ (Figure 7O). Furthermore, knocking down SIRT5 using siRNAs could not affect the ubiquitination of SUCLG2^K93R^ (Figure S7I, Supporting Information). The overexpression or knockdown of SIRT5 also did not change the degradation rate of SUCLG2^K93R^ (Figure S7J and K, Supporting Information). Taken together, these results demonstrated that SIRT5 is the desuccinylase for SUCLG2 on Lys93 and facilitates the TRIM21‐mediated degradation of SUCLG2.

### 2.8 Inhibiting the Succinylation of SUCLG2 on K93 Leads to the Mitochondrial Dysfunction and Reduced Tumorigenesis of LUAD Cells

**Figure 8:**
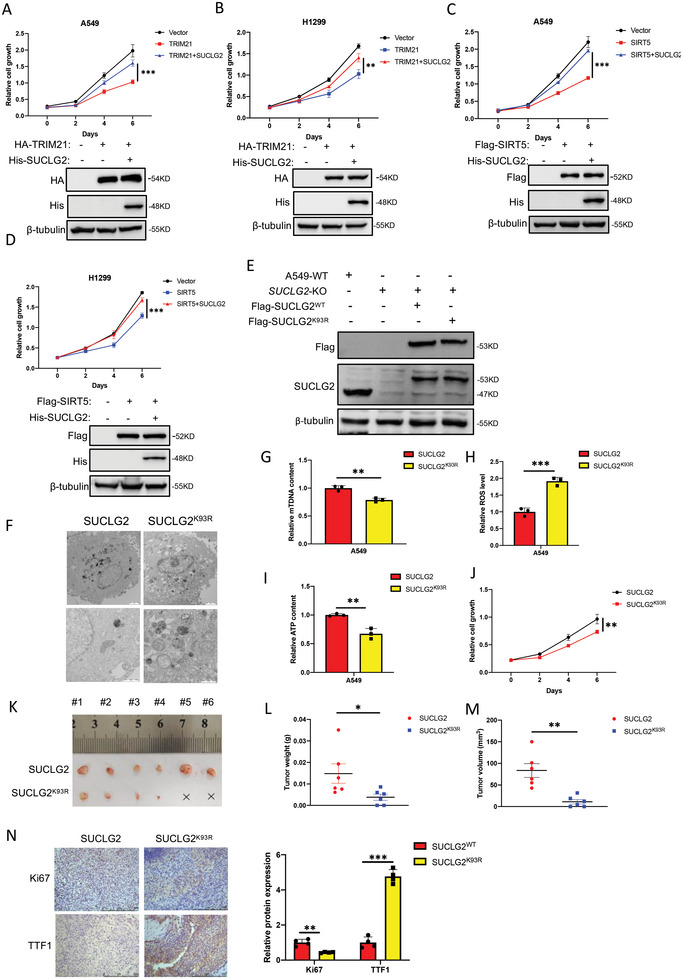
Inhibition of succinylation at the SUCLG2 K93 site leads to the mitochondrial dysfunction and reduced tumorigenesis of LUAD cells. A and B) LUAD cell lines A549 (A) and H1299 (B) were transfected with His‐SUCLG2 and HA‐TRIM21 plasmids. Twenty‐four hours later, cells were seeded in 24‐well plates. At the indicated times, cells were fixed with 4% paraformaldehyde and stained with 1% crystal violet. The dye was extracted with 10% acetic acid, and the relative proliferation was assessed based on the increase in absorbance at 595 nm (upper panels). Western blotting was used to determine the overexpression efficiency (bottom panels). The data represent the average of three independent experiments (mean ± SD). **p < 0.01, ***p < 0.001. C and D) LUAD cell lines A549 (C) and H1299 (D) were transfected with His‐SUCLG2 and Flag‐SIRT5 plasmids. Cell growth assays were performed. (upper panels). Western blotting was used to determine the overexpression efficiency (bottom panels). The data represent the average of three independent experiments (mean ± SD). ***p < 0.001. E) A549‐SUCLG2^WT^ and A549‐SUCLG2^K93R^ cell lines were constructed by CRISPR‐Cas9. The expression of the SUCLG2 protein was detected by western blotting. F) Mitochondrial morphology was observed using TEM. Scale bars: 2 µm (upper panels) and 1 µm (bottom panels). G) qPCR analysis of mtDNA content in A549‐SUCLG2^WT^ and A549‐SUCLG2^K93R^ cells. Data represent the average of three independent experiments (mean ± SD). **p < 0.01. H) ROS generation was assessed with 5 µm H2DCFDA added to A549‐SUCLG2^WT^ and A549‐SUCLG2^K93R^ cells, followed by confocal microscopy. The data represent the average of three independent experiments (mean ± SD). ***p < 0.001. I) The intracellular ATP concentration was determined in A549‐SUCLG2^WT^ and A549‐SUCLG2^K93R^ using luminescence values and normalized to the protein content in each sample. The data represent the average of three independent experiments (mean ± SD). **p < 0.01. J) A549‐SUCLG2^WT^ and A549‐SUCLG2^K93R^ cells (5 × 10^3^) were seeded in 24‐well plates. At the indicated times, the cells were fixed with 4% paraformaldehyde and stained with 1% crystal violet. The dye was extracted with 10% acetic acid, and the relative proliferation was assessed based on the increase in absorbance at 595 nm. The data represent the average of three independent experiments (mean ± SD). **p < 0.01. K–M) A549‐SUCLG2WT and A549‐SUCLG2K93R cells were subcutaneously injected into the flanks of nude mice (six mice per group). Twenty‐eight days later, the tumors were dissected out and photographed (K). The weight (L) and volume (M) of xenograft tumors were measured. The data represent the average of six independent experiments (mean ± SD). **p < 0.01, *p < 0.05. N) Representative IHC images of xenograft tumors with anti‐Ki67 and anti‐TTF1 (left‐hand panels). Quantification of the IHC staining was performed using ImageJ software (right‐hand panel), and the results represent the average of four independent experiments (mean ± SD). **p < 0.01, ***p < 0.001.

To further examine whether TRIM21 affects tumorigenesis by regulating the ubiquitination and degradation of SUCLG2, we overexpressed TRIM21 and SUCLG2 in A549 and H1299 cells, and the cell growth assays revealed that overexpression of TRIM21 inhibited the growth of A549 and H1299 cells. However, SUCLG2 expression overcame the inhibition on cell proliferation caused by overexpressing TRIM21 (**Figure** **8**A,B). Similarly, SIRT5 also inhibit the growth of A549 and H1299 cells, and SUCLG2 reduced the inhibitory effects caused by overexpressing SIRT5 (Figure 8C and D). These results indicated that SUCLG2 mediated the functions of SIRT5 and TRIM21 on cell proliferation in LUAD cells. To provide a theoretical basis for developing anti‐tumor drugs by targeting SUCLG2, we depleted endogenous SUCLG2 and stably reintroduced wild‐type SUCLG2 (SUCLG2^WT^) or the SUCLG2^K93R^ mutant into A549 cells (Figure 8E). We assessed the effects of the SUCLG2^K93R^ mutant on mitochondrial function and used TEM to observe the morphological changes of the mitochondria. The mitochondria in A549‐SUCLG2^WT^ cells presented intact cristae and normal morphological characteristics. However, the mitochondria in A549‐SUCLG2^K93R^ cells appeared vacuolized and swollen with fractured cristae (Figure 8F). To further define the difference in mitochondrial quality, we detected the mtDNA copy number in A549‐SUCLG2^WT^ and A549‐SUCLG2^K93R^ cells. We found that mtDNA copy number was decreased in A549‐SUCLG2^K93R^ cells compared with A549‐SUCLG2^WT^ cells (Figure 8G). We also found a significant accumulation of ROS in A549‐SUCLG2^K93R^ cells compared with A549‐SUCLG2^WT^ cells (Figure 8H). The ATP content was higher in A549‐SUCLG2^WT^ cells than in A549‐SUCLG2^K93R^ cells (Figure 8I). Our findings indicated that mitochondrial function was better in A549‐SUCLG2^WT^ than in A549‐SUCLG2^K93R^. We next performed a cell proliferation assay in A549‐SUCLG2^WT^ and A549‐SUCLG2^K93R^ cells. The results showed that the SUCLG2^K93R^ mutant suppressed cell proliferation (Figure 8J). We then assessed the tumorigenesis of A549‐SUCLG2^K93R^ cells in a xenograft model. Xenografts of A549‐SUCLG2 cells displayed a reduced tumor weight and volume compared with those of A549‐SUCLG2^WT^ (Figure 8K–M), and the IHC results demonstrated that the expression of Ki67 was decreased and that of TTF1 was increased in SUCLG2^K93R^ mutant tumors compared with SUCLG2^WT^ tumors (Figure 8N). These results demonstrated that inhibiting the succinylation of SUCLG2 on K93 leads to the mitochondrial dysfunction and reduced tumorigenesis of LUAD cells.

## 3 Discussion

SUCLG2 and SUCLA2 are different β subunits of SUCL, which determine the substrate specificity of the enzyme. The distribution of these two proteins is different, with SUCLG2 mainly located in the liver and kidneys and SUCLA2 mainly in the brain and skeletal muscle.^[^ ^25^ ^]^ The functions of SUCLG2 and SUCLA2 in cancer are gradually being recognized by researchers. SUCLG2 knockdown suppressed NE differentiation in prostate cancer cells and reduced tumor growth in a xenograft model.^[^ ^3^ ^]^ SUCLA2 deletion induced a significant metabolic shift, including lower mitochondrial respiratory activity.^[^ ^26^ ^]^ However, it is currently unclear whether there is a functional difference between the two proteins in cancer progression. In this study, we demonstrated that the protein expression of SUCLG2 was significantly upregulated in LUAD cell lines and tissues, and SUCLG2 depletion reduced the proliferation of LUAD cells. However, the expression of SUCLA2 was not upregulated in LUAD, and SUCLA2 knockout did not affect the proliferation of LUAD cells. This suggested that SUCLG2, but not SUCLA2, plays an important role in LUAD. Thus, the functions of SUCLA2 and SUCLG2 differ depending on the cancer type.

Metabolic reprogramming in cancer cells has been intensively studied in past decades. Mitochondrial dysfunction is an important factor in tumor metabolic reprogramming.^[^ ^27^ ^]^ In recent years, an increasing number of studies have shown that mitochondrial dysfunction exists in tumor cells and affects the occurrence and development of tumors. Succinylation is an important post‐translational modification that is closely related to mitochondrial dysfunction. Multiple studies have reported that mitochondrial functions are regulated by protein succinylation. For example, SIRT5 plays a major role in the maintenance of mitochondrial homeostasis. The knockdown of SIRT5 led to a reduction in the ETC complex and mitochondrial dysfunction via an increase in the succinylation of apoptosis‐inducing factor 1 (AIFM1) in rat nucleus pulposus (NP) cells.^[^ ^28^ ^]^ In addition, SUCLA2 enhanced the K311 succinylation of glutaminase liver isoform (GLS) and increased its enzymatic activity. Activated GLS increased glutaminolysis and upregulated mitochondrial function in prostate cancer cells.^[^ ^29^ ^]^ In our study, we demonstrated that SUCLG2 knockout led to mitochondrial dysfunction through an increase in the succinylation of mitochondrial proteins and inhibited the function of key metabolic enzymes. Compared with other studies, our work revealed that SUCLG2 affected mitochondrial functions by regulating the global succinylation levels of multiple proteins rather than those of a single protein. Therefore, SUCLG2 not only acts as a metabolic enzyme but also as a key regulatory factor for succinylation that affects LUAD progression.

TRIM21 belongs to the tripartite motif (TRIM) family and is an important E3 ligase. TRIM21 has been extensively studied in tumors and regulates the degradation of many cancer‐related proteins, like P53 and interferon regulatory factor 5 (IRF5).^[^ ^30^, ^31^ ^]^ TRIM21 is also reported to regulate tumor metabolism. For example, TRIM21 directly interacted with SHMT2 and ubiquitinated SHMT2 via K63‐linkage to inhibit colon cancer cell proliferation and tumor growth.^[^ ^32^ ^]^ However, more studies are needed to further clarify its role in tumor metabolism. The mechanism of SUCLG2 degradation under physiological conditions has not been investigated. In this study, we found that TRIM21 promoted the K63‐linkage ubiquitination and lysosome pathway degradation of SUCLG2. We also elucidated that the K200 of SUCLG2 was the key ubiquitination site for TRIM21. As SUCLG2 regulates global succinylation in LUAD, this indicates that TRIM21 may have more extensive functions, for example, regulating succinylation, depending on its substrates. Therefore, our understanding of TRIM21 in cancer should not only be limited to its E3 ubiquitin ligase function but also focus on its substrate function.

As the most important desuccinylase, SIRT5 plays an important role in regulating protein function. SIRT5 is mainly located in mitochondria, and researchers found that the targets of SIRT5 included 100% of proteins in ketogenesis, 93% of proteins in β‐oxidation, 58% of proteins in amino acid catabolism, 43% of proteins in the TCA cycle, and 43% of proteins in ATP synthesis.^[^ ^23^, ^33^ ^]^ SUCLG2 is localized in the mitochondrial matrix. Our results showed that SIRT5 directly interacted with SUCLG2 and desuccinylated SUCLG2. This indicates that SIRT5‐mediated succinylation regulation is mediated not only by its direct binding to the desuccinylation substrate but also by the regulation of SUCLG2 expression to influence overall succinylation in cancer cells. However, we did not observe similar correlation between the protein expression of SIRT5 and SUCLG2, like that between TRIM21 and SUCLG2, in lung cancer tissues and adjacent normal tissues. We thought that TRIM21 was the E3 ligase that directly regulated the degradation of SUCLG2, thus their expressions showed the negative correlation. But SIRT5 was the desuccinylase acting on multiple substrates and its effects on SUCLG2 degradation was indirect, needing TRIM21 to act as the direct degradation factor. SIRT5 mediated the desuccinylation of SUCLG2 on Lys93, and TRIM21 was more inclined to bind SUCLG2 under this state, this increased TRIM21‐mediated ubiquitination of SUCLG2 on Lys200. Thus, desuccinylation of SUCLG2 on Lys93 is like a switch that activates the ubiquitination and protein degradation of SUCLG2.

Mitochondrial succinylation is crucial and a widespread regulatory mechanism for mitochondrial proteins. We found that SUCLG2 knockout promoted the succinylation of proteins by up‐regulating the concentration of the succinyl‐CoA, leading to the inhibition of mitochondrial proteins. Thus, SUCLG2 inhibition dampened mitochondrial function through succinylation and inhibited tumorigenesis. Meanwhile, SUCLG2 was also succinylated which increased the protein stability of SUCLG2, decreased the succinylation of mitochondrial proteins and promoted the mitochondrial function. This feedback loop of SUCLG2 plays an important role in maintain mitochondrial function.

Taken together, our study elucidates the role of SUCLG2 in mitochondrial dysfunction and clarifies the mechanism of the succinylation‐mediated protein homeostasis of SUCLG2 in LUAD, thereby providing a theoretical basis for developing anti‐tumor drugs by targeting SUCLG2.

## 4 Experimental Section

### Antibodies and Reagents

Antibodies: The mouse monoclonal antibodies against the His tag (MA1‐21315, 1:5000), HA (26 183, 1:3000), and ubiquitin (14‐6078‐82, 1:1000) were purchased from Thermo Fisher Scientific. The rabbit polyclonal antibodies against SUCLG2 (14240‐1‐AP, 1:2000), SUCLA2 (12627‐1‐AP, 1:1000), IDH2 (15932‐1‐AP, 1:1000), MDH2 (15462‐1‐AP, 1:1000), ME2 (24944‐1‐AP, 1:1000), and TRIM21 (12108‐1‐AP, 1:2000); the mouse monoclonal antibodies against β‐tubulin (66240‐1‐Ig, 1:5000), SIRT5 (67257‐1‐Ig, 1:1000), ACOT9 (66532‐1‐lg, 1:5000), β‐actin (60008‐1‐Ig, 1:4000), GAPDH (60004‐1‐Ig, 1:3000), the Flag tag (66008‐4‐Ig, 1:3000), and VDAC1 (66345‐1‐Ig, 1:2000) were ordered from Proteintech. The rabbit polyclonal antibodies against the K63‐linkage‐specific polyubiquitin antibody (5621, 1:1000) and the K48‐linkage‐specific polyubiquitin antibody (8081, 1:1000) were obtained from Cell Signaling Technology. The mouse monoclonal antibody against the SQSTM1/p62 monoclonal antibody (TA502127, 1:2000) was ordered from OriGene. The anti‐succinyllysine rabbit polyclonal antibody (PTM‐401, 1:500) and the anti‐acetyllysine mouse monoclonal antibody (PTM‐101, 1:1000) were ordered from PTM BIO.

Reagents: Chloroquine (C7698) and cycloheximide (C6628) were ordered from Sigma. MG132 (C1791) was purchased from Biovision. Polyformaldehyde (P1110, 4%) was purchased from Solarbio. *SUCLG2* siRNA (SR305801), *SIRT5* siRNA (SR323578), and *TRIM21* siRNA (SR321894) were ordered from Origene.

### Plasmids

PCR‐amplified SUCLG2, ME2, GAPDH, ACOT9, IDH2, MDH2, TRIM21, p62, and SUCLA2 were cloned into the pcDNA‐His/v5, pCDH‐Flag, pCMV‐Flag, and pCMV‐HA vectors. SUCLG2 (K78R), SUCLG2 (K93R), SUCLG2 (K338R), SUCLG2 (K101R), SUCLG2 (K118R), SUCLG2 (K132R), SUCLG2 (K200R), and SUCLG2 (K386R) mutation plasmids were generated using the ClonExpress II One Step Cloning kit (Vazyme). Plasmids overexpressing SIRT5 and SIRT7 were donated by Professor Hongquan Zhang. The primer sequences are shown in Table S3 (Supporting Information).

### Cell Culture and Transfection

Cell culture: The human lung epithelial cell line BEAS‐2B was purchased from the National Collection of Authenticated Cell Cultures, LUAD cell lines (A549, H1299, H23, H292, HCC827, H358, PC9, and H1975) were purchased from the cell library of the Chinese Academy of Sciences, and SPC‐A1 was purchased form BLUEFBIO. Cells were cultured in Roswell Park Memorial Institute (RPMI) 1640 medium (Gibco, C11875500BT) supplemented with 10% fetal bovine serum (FBS; SORFA). All cells were cultured under an atmosphere of 5% CO2 at 37 °C.

Transfection: Cells at 70%–80% confluence were transfected with the indicated plasmids using the SuperFectin DNA Transfection Reagent kit (Pufei, 2103–100). siRNA transfection was performed with siTran 2.0 siRNA transfection reagent (OriGene, TT320002) when cell confluence reached 50%. All transfection experiments were performed according to the instruction manual.

### Patient Samples

Fresh LUAD tissues and adjacent normal tissues were obtained from the First Affiliated Hospital of Nanchang University with approval from the Medical Research Ethics Committee of the First Affiliated Hospital of Nanchang University (2020) CDYFYYLK (1‐19). All samples were collected with the patients’ informed consent and frozen and preserved at −80 °C for subsequent experimental analysis.

### Cell Proliferation Assay

Cell growth assay: A total of 3000–5000 cells in RPMI 1640 with 10% FBS were seeded in 24‐well plates, and each group of cells had three replicates. At the specified time, cells were fixed in 4% formaldehyde at room temperature for 15 min before being stained with 1% crystal violet. Then, cells were incubated with 10% acetic acid, and the relative proliferation was determined by measuring the absorbance at 595 nm.

Colony formation assay: A total of 500 cells in RPMI 1640 with 10% FBS were seeded in 6‐well plates and cultured for 9 days before staining with 1% crystal violet. Thereafter, the colonies were counted and photographed.

### RT‐PCR

Cell total RNA was extracted using the TRTGene reagent (Genestar P118‐05), and 1 µg total RNA was used for reverse transcription to cDNA using the Prime Script RT reagent kit with gDNA Eraser (Takara, RR047A). qRT‐PCR was performed with the SYBR Green Premix Ex Taq II kit (Takara, RR820A). The data were normalized to the expression of the control gene *GAPDH* for each experiment. All gene reactions were performed in triplicate. All gene primers used for RT‐PCR are presented in Table S3 (Supporting Information).

### Immunoprecipitation and Western Blotting

Immunoprecipitation assay: Cells were washed with phosphate‐buffered saline (PBS) three times in 5 min and lysed with NP‐40 buffer (150 mM NaCl, 20 mM β‐glycerol phosphate, 1 mM Na orthovanadate, 20 mM NaF, 0.5% Nonidet P‐40, 20 mM Hepes, pH 7.4) with PMSF for 30 min at 4 °C. Then, the cell lysates were collected, and Protein G‐Agarose (Roche, 11 243 233 001) was added along with the indicated antibodies, with overnight incubation at 4 °C. The samples were washed with PBS three times and centrifuged at 3000 g for 3 min at 4 °C between each wash. The samples were added to 2 × loading buffer and boiled for 10 min.

Western blotting: The samples were added to 10% SDS‐PAGE and transferred to PVDF membranes (Millipore, IPVH00010), then blocked with 5% skim milk (Solarbio D8340) for 1 h at room temperature. After incubation with the indicated primary antibodies, the membranes were washed with TBS'T three times in 10 min and incubated with the secondary antibody for 1 h at room temperature. The membranes were washed three times with TBS'T and stained with an ECL detection reagent (TIANGEN, PA112‐01). Digital gel image analysis (TANON 5500) was used to visualize the proteins.

### Transwell Migration Assay and Scratch Wound Healing Assay

Transwell migration assay: A total of 50 000–100 000 tumor cells were seeded inside transwell inserts (8 µm pore size) containing 0.2 ml RPMI 1640 with 1% FBS. As a chemoattractant, 500 µl RPMI 1640 with 10% FBS was filled in the lower chamber of the transwell device. At the indicated times, cells translocated to the lower surface of the filters were fixed in 4% formaldehyde and then stained with 1% crystal violet. Cells were counted using a light microscope (Olympus, IX71).

Scratch wound healing assay: Tumor cells were seeded inside 6‐well plates containing 2.0 ml RPMI 1640 with 10% FBS. A straight line was drawn across the cell layer using a 200 µl pipet tip when the cells formed a monolayer of 80%–90% confluence. PBS was used to wash the cells three times and fresh medium with 1% FBS was added to the cells. At the indicated times, the samples were photographed under a light phase‐contrast microscope.

### CRISPR‐Cas9‐Mediated Gene Disruption

The sgRNA sequences targeting SUCLG2 were designed by the CRISPR designer at https://chopchop.cbu.uib.no/. The sgRNA sequences were cloned into the lentiCRISPR‐v2‐Flag vector. A total of 5 × 10^5^ HEK‐293T cells were seeded in a 6‐well plate. After 24 h, the cells were transfected with the packaging construct pSPAX2 (2 µg), lentiviral vector pMD2.G (1.5 µg), and lentiCRISPR‐v2‐Flag‐SUCLG2 (1 µg) using Lipofectamine 2000. At the indicated times, the virus‐containing cell culture supernatant was collected, filtered with a 0.45 µm filter, and subsequently used to infect A549 or H1299 cells. At 24 h after infection, the cells were selected with 10 µg mL^−1^ of puromycin (Solarbio, P8230). The cells were counted and cultured individually in 96‐well plates. At indicated times, SUCLG2‐KO and SUCLA2‐KO stable cell lines were identified by western blotting. A control CRISPR‐Cas9 plasmid was used as a negative control.

### Immunofluorescence Assay

A total of 1 × 10^3^ cells were seeded in a 24‐well plate with a cell slide (NEST 801 010) and cultured for 24 h. The cell slides were washed with PBS three times in 5 min, then fixed with 4% paraformaldehyde for 30 min. The cells were washed three times with PBS, then sealed with a blocking buffer (3% BSA + 0.2% Trition‐X‐100 in PBS) for 1 h at room temperature, following which the primary antibodies were added with overnight incubation. The cells were washed with wash buffer (0.2% BSA + 0.05% Trition‐X‐100 in PBS) five times in 15 min, and fluorescent secondary antibodies were added with 1 h of incubation. The cells were washed three times with wash buffer, and then DAPI (Southern Biotech 0100–20) was added to the cells for staining. Finally, a laser confocal microscope (ZEISS) was used for photography.

### In Vivo Xenograft Assay

4‐week‐old male BALB/c‐Nude mice were purchased from Gempharmatech Co., Ltd (Jiangsu, China). All nude mice were lived in the SPF animal facility of the Institute of Translational Medicine at Nanchang University and experimental procedures were approved by the Institutional Animal Use and Care Committee of Nanchang University (NCULAE‐20221130009). All animal experiments were performed according to the established guidelines. The study complied with all the relevant ethical regulations on animal research.

A549 cells (1 × 10^7^) stably expressing the control CRISPR‐Cas9 plasmid or SUCLG2 knockout or SUCLG2‐depleted A549 cells rescued with SUCLG2‐WT or SUCLG2^K93R^ were injected subcutaneously into the flanks of 4‐week‐old male BALB/C nude mice. After inoculation for 4 weeks, the mice were euthanized, and the tumors dissected out. Tumor volume (calculated as π/6 × [large diameter] × [smaller diameter]^2^) and weight were measured.

### IHC

Mouse tumors were dissected and fixed with 4% paraformaldehyde. Samples were sent to the Servicebio company for IHC. The tissue sections from tumors were stained with anti‐Ki67 (Abcam, ab15580), anti‐TTF1 (Abcam, ab76013), and anti‐SUCLG2 antibodies. Micrographs were obtained using an Olympus IX71 microscope.

### mtDNA Extraction and Analysis

The total mtDNA of cells was extracted with the Universal Genomic DNA Purification Mini Spin Kit (Beyotime, D0063). The quantity of mtDNA was determined by RT‐PCR as previously described. All gene primers used for RT‐PCR are shown in Table S3 (Supporting Information). The relative mtDNA content was calculated by the formula mtDNA = 2 × 2^(CtnDNA–CtDNA)^, where Ct_mt_DNA and Ct_n_DNA refer to the threshold cycles of mtDNA and nuclear (n)DNA.

### Detection of Intracellular ROS

A total of 1 × 10^4^ cells were seeded in a 24‐well plate and cultured for 24 h. Then, the cells were incubated with H2DCFDA (20 µm; MedChemExpress, HY‐D0940) for 30 min in the dark at room temperature. After washing with PBS three times, the fluorescence intensity of the cells was detected at Ex/Em = 488/525 nm. Ten cells were randomly selected, and the fluorescence intensity was analyzed with Image J.

### ATP Production Measurement

The ATP concentration in LUAD cells was determined using an ATP assay kit (Beyotime, S0026). Cells were transfected with either individual siRNAs targeting SUCLG2 or control siRNAs. Forty‐eight hours later, cells were lysed and centrifuged at 12 000 *g* for 5 min at 4 °C. Then, the lysed cells were added to the reaction buffer according to the manufacturer's instructions. The intracellular ATP concentration was determined based on the luminescence values and normalized to the protein content in each sample.

### Mitochondria and Cytosol Extraction

A total of 1 × 10^7^ cells were seeded in a 10 cm dish and cultured for 24 h. Then, the cells’ mitochondria and cytosol were extracted using the Qproteome Mitochondria Isolation Kit (QIAGEN, 37 612).

### Measurement of MDH2 Activity

MDH2 activity was measured using the NADP‐Malate Dehydrogenase (NAD‐MDH) Activity Assay Kit (Beijing Solarbio Science & Technology Co., Ltd.) Briefly, cells were subjected to ultrasound and 5 µl supernatant was added to 195 µl MDH buffer. The absorbance at 340 nm was measured with a microplate reader (A1). After 1 min, the absorbance at 340 nm was measured again (A2). The formula △A = (A1 − A2) was used to determine MDH activity.

### Measurement of IDH2 Activity

IDH2 activity was measured using the Isocitrate Dehydrogenase Mitochondrial (ICDHm) Activity Assay Kit (Beijing Solarbio Science & Technology Co., Ltd.) according to the manufacturer's protocol.

### Measurement of ME2 Activity

ME2 activity was measured using the NADP Malic Enzyme (NADP‐ME) Activity Assay Kit (Beijing Solarbio Science & Technology Co., Ltd.) according to the manufacturer's protocol.

### Measurement of GAPDH Activity

GAPDH activity was measured using the GAPDH Enzyme Activity Assay Kit (Sigma, MAK277) according to the manufacturer's protocol.

### Untargeted Metabolomics

Untargeted metabolomics was performed and analyzed by Novogene.

### Succinyl 4D Mass Spectrometry Analysis

Succinyl 4D mass spectrometry analysis was performed and analyzed by PTM BIO.

### Statistical Analysis

GraphPad Prism 8 software (version 8.3.1) was used for statistical analysis. The mean values obtained for the control and experimental groups were analyzed for significant differences. All data are presented as mean ± SD. Student's t‐test was used for statistical evaluation. Statistical significance was reported follows, ns, not significant, *p* > 0.05; *p < 0.05; **p < 0.01; ***p < 0.001.

## Conflict of Interest

The authors declare no conflicts of interest.

## Author Contributions

J.W. and T.H. designed the study and Q.H. implemented the design in practice. Q.H., J.X., L.W., Y.Y., R.L., K.W., and T.Z. designed and completed the experiments. Y.W. and M.G. collected clinical tissues. T.H. and Q.H. analyzed the data and wrote the manuscript. J.W. and T.H. revised the paper. This manuscript was approved by all authors.

## Supporting information

Supporting Information

Supporting Information

Supporting Information

## Data Availability

The data that support the findings of this study are available from the corresponding author upon reasonable request.
