# Supplementary material for: SUCLG2 Regulates Mitochondrial Dysfunction through Succinylation in Lung Adenocarcinoma
Source: Adv Sci (Weinh). 2023 Oct 30;10(35):2303535. doi: 10.1002/advs.202303535 (PMC10724390; doi:10.1002/advs.202303535)
Supplement: Supplementary file 1 — Supporting Information [file ADVS-10-2303535-s003.pdf]

ADVANCED SCIENCE
Open Access

Supporting Information

for *Adv. Sci.*, DOI 10.1002/advs.202303535

SUCLG2 Regulates Mitochondrial Dysfunction through Succinylation in Lung Adenocarcinoma

Qifan Hu, Jing Xu, Lei Wang, Yi Yuan, Ruiguang Luo, Mingxi Gan, Keru Wang, Tao Zhao, Yawen Wang, Tianyu Han and Jian-Bin Wang**

Supplementary Information

S1

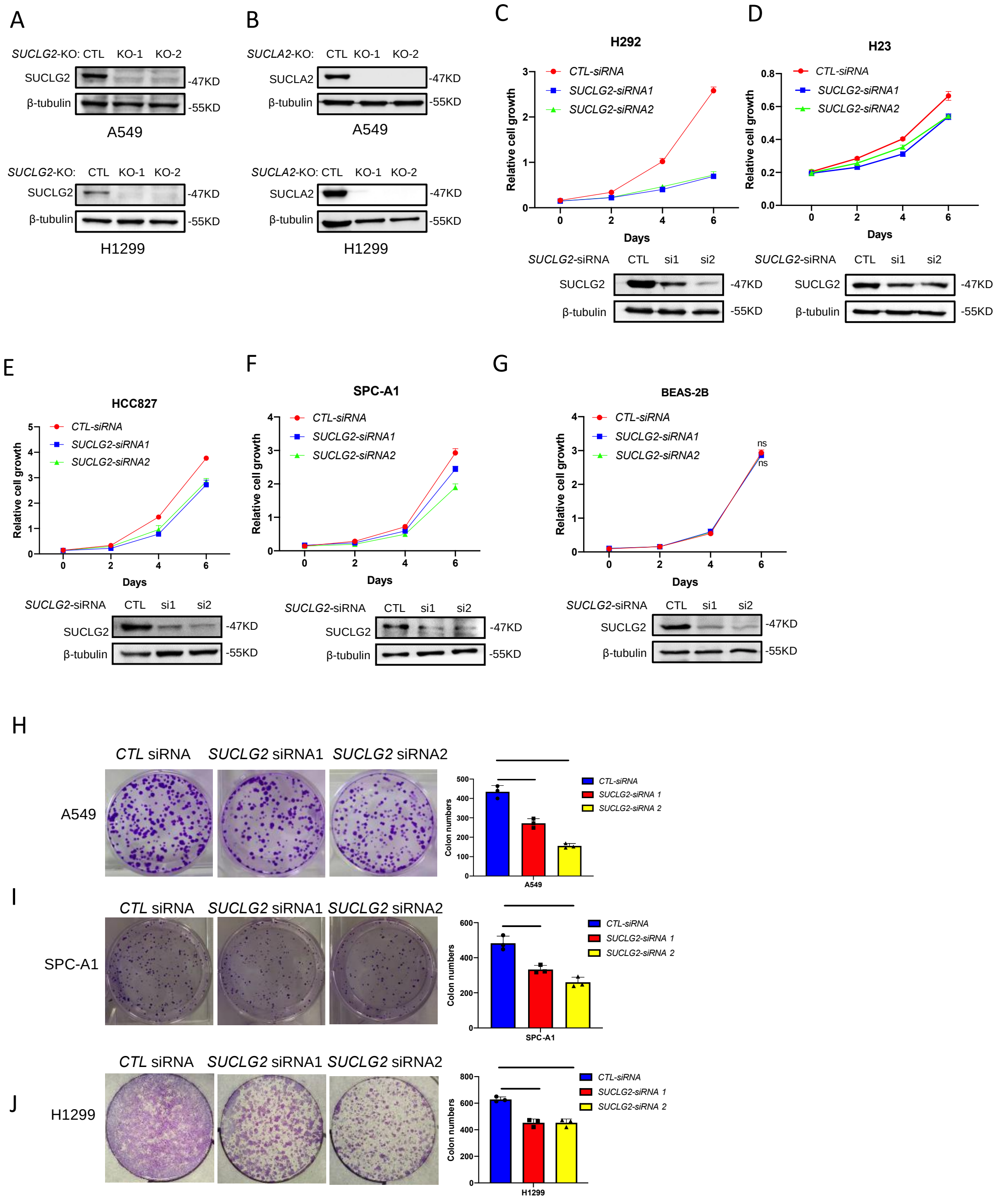

Figure S1. SUCLG2 knockdown attenuates the proliferation and tumorigenicity of LUAD cells. (A-B) The effects of knockout of SUCLG2 (A) and SUCLA2 (B) in A549 and H1299 cells were detected by western blotting. (C-G) LUAD cell lines H292 (C), H23 (D), HCC827 (E), and SPC-A1 (F) and the human lung epithelial cell line BEAS-2B (G) were transfected with either individual siRNAs targeting SUCLG2 or control siRNAs. Twenty-four hours later, cells were seeded in 24-well plates. At

the indicated times, cells were fixed with 4% paraformaldehyde and stained with 1% crystal violet. The dye was extracted with 10% acetic acid, and the relative proliferation was assessed based on the increase in absorbance at 595 nm (upper panels). Western blotting was used to determine the knockdown efficiency (bottom panels). The data represent the average of three independent experiments (mean ± SD). ***P < 0.001, **P < 0.01, ns: P > 0.05. (H-J) LUAD cell lines A549 (H), SPC-A1 (I), and H1299 (J) were transfected with either individual siRNAs targeting SUCLG2 or control siRNAs. A colony formation assay (left-hand panels) was performed and quantitatively analyzed using ImageJ software. The results represent the average of three independent experiments (mean ± SD). **P < 0.01, ***P < 0.001 (right-hand panels).

S2

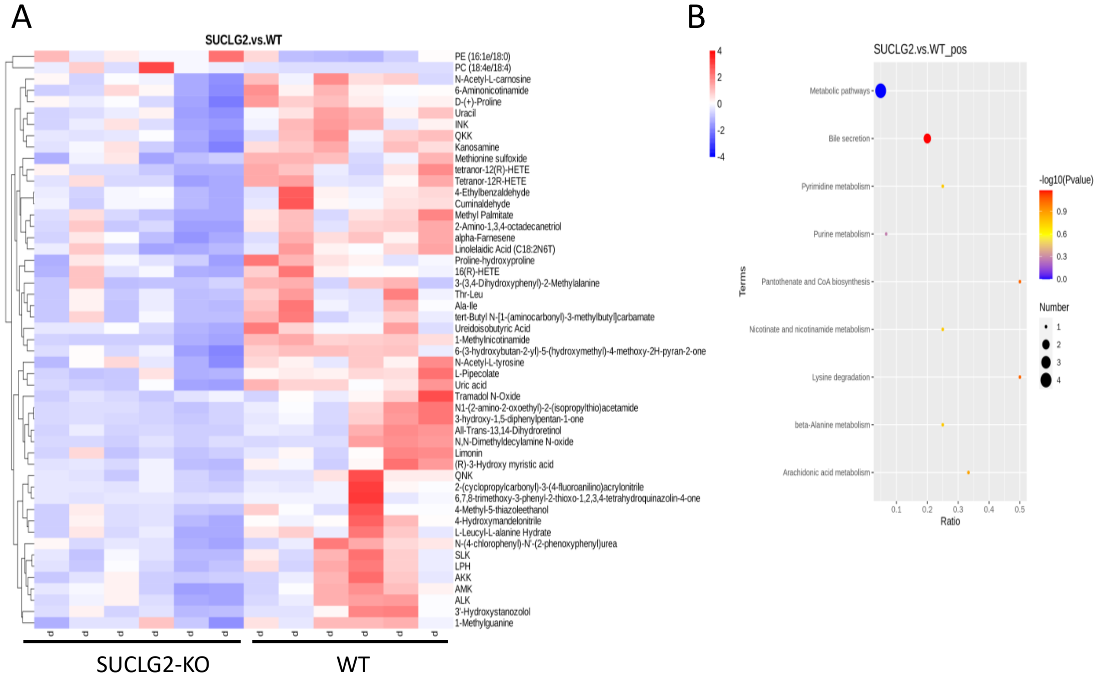

Figure S2 SUCLG2 knockout affecting the metabolism of LUAD cells. (A and B) A549-SUCLG2-KO and A549-WT cells were used for untargeted positive ion mode

metabolomics analysis (A). The positive ion mode metabolomics relative pathway analysis is shown (B). n = 6 per group.

S3

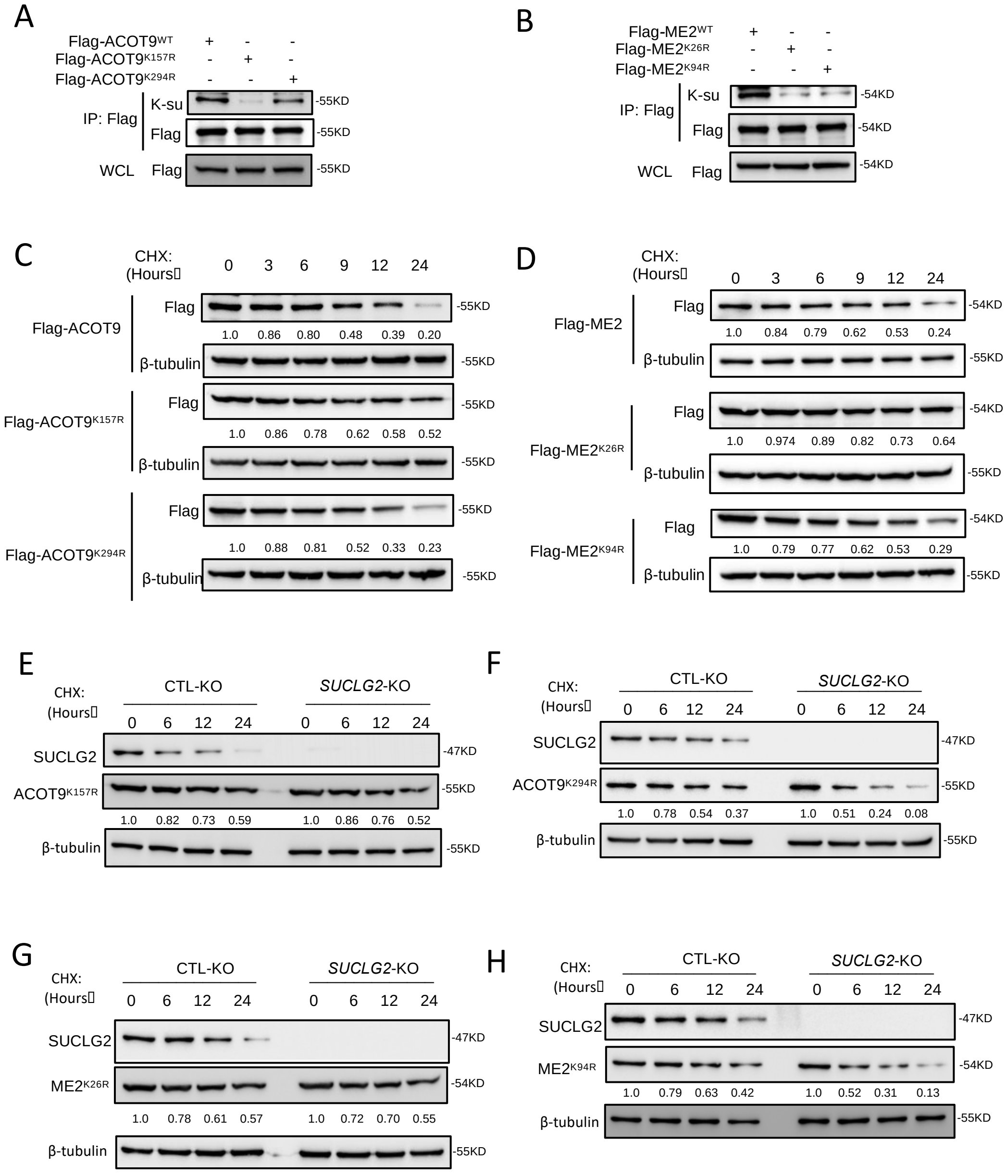

Figure S3. Succinylation affects the degradation of ACOT9 and ME2. (A and B) The indicated plasmids were transfected into A549 cells. Co-IP and western blot were used to detect the succinylation level of ACOT9 (A) and ME2 (B). (C and D) A549 cells were transfected with the indicated plasmids, then treated with CHX (20 µg/mL) for 0h, 3h, 6h, 9h, 12h, and 24 h. The degradation rate of ACOT9 (C) and ME2 (D)

was detected by western blotting. (E-G) A549-WT and A549-SUCLG2-KO cells were transfected with the indicated plasmids, then treated with CHX (20 μg/mL) for 0h, 6h, 12h and 24 h. The degradation rate of ACOT9 and ME2 mutant proteins were detected by western blotting.

S4

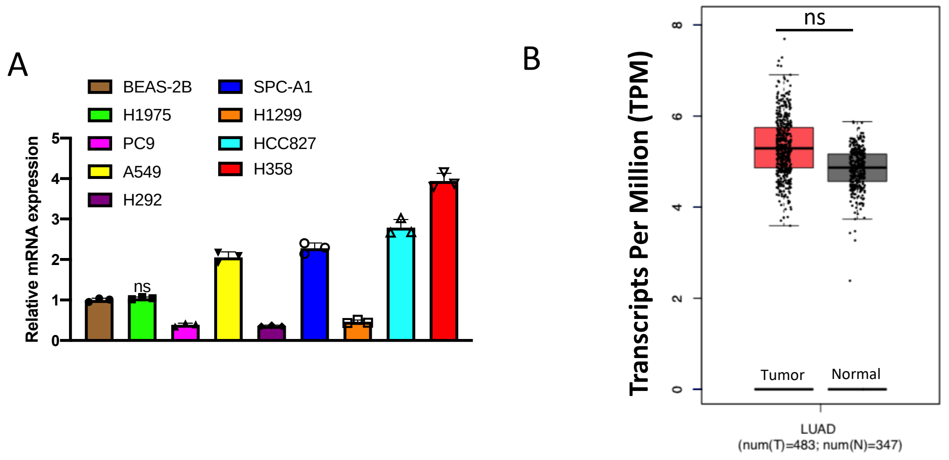

Figure S4. The mRNA level of SUCLG2 is not significantly upregulated in LUAD. (A) The mRNA level of SUCLG2 was detected in LUAD cell lines and the normal epithelial cell line BEAS-2B with RT-PCR. The data represent the average of three independent experiments (mean ± SD). ns, $P > 0.05$; ***$P < 0.001$. (B) SUCLG2 TPM expression in LUAD tissues and adjacent tissues was analyzed by GEPIA (http://gepia.cancer-pku.cn).

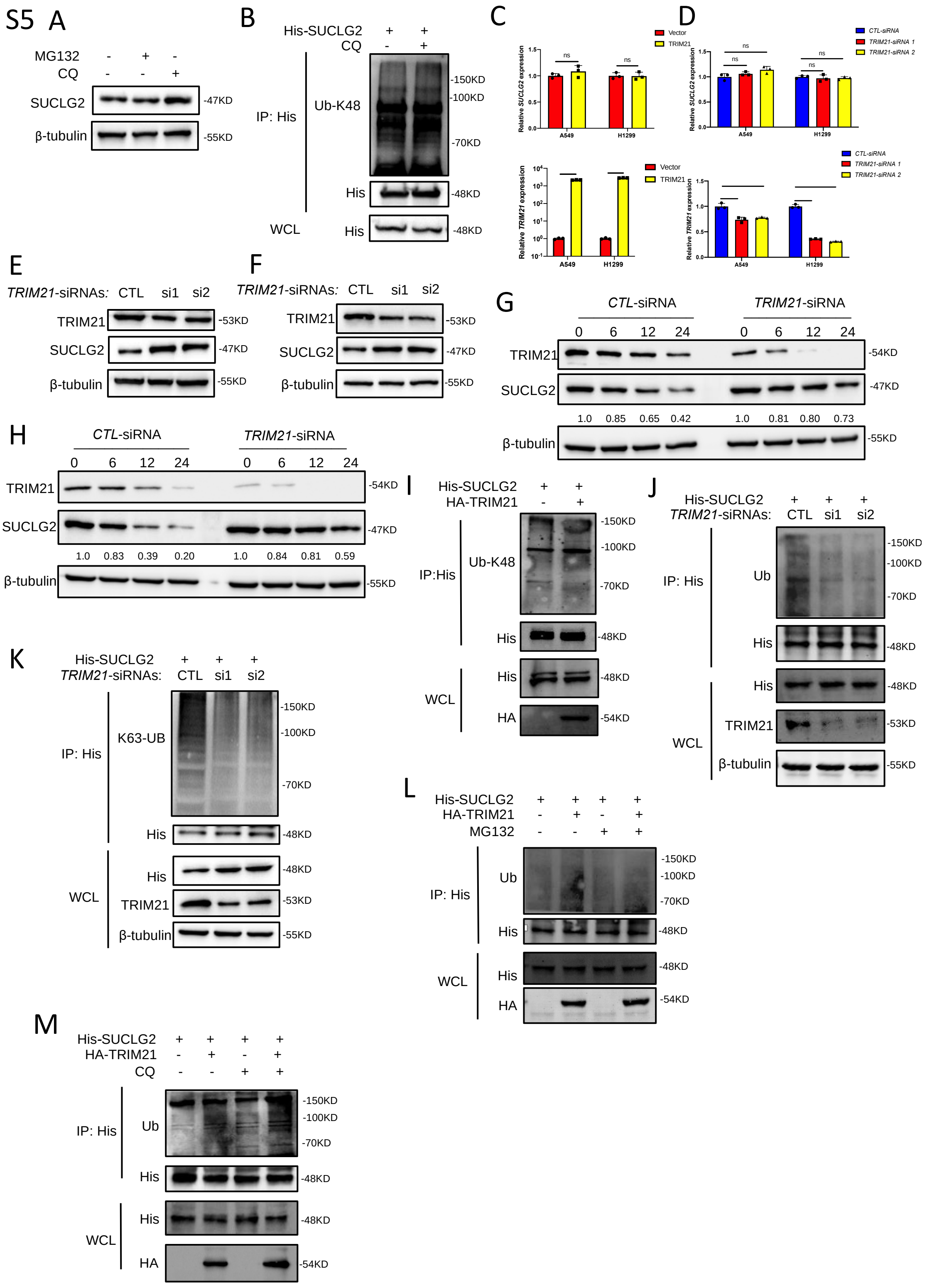

Figure S5. TRIM21 promotes the ubiquitination and degradation of SUCLG2. (A) CQ (20 μM) or MG132 (20 μM) was added to BEAS-2B cells for 24h. The expression of SUCLG2 was detected using western blotting. (B) The

indicated plasmids were transfected into A549 cells treated with CQ (20 μM) for 24h. Co-IP was used to detect the K48-linkage ubiquitination level of SUCLG2. (C and D). In A549 and H1299 cells, TRIM21 was overexpressed (C) or knocked down (D) and SUCLG2 mRNA was detected by RT-PCR (upper panel). RT-PCR was used to determine the overexpression or knockdown efficiency of TRIM21 (bottom panels). The data represent the average of three independent experiments (mean ± SD). ns, $P > 0.05$; ***$P < 0.001$. (E and F) The expression of SUCLG2 was detected by western blotting in A549 (E) and BEAS-2B (F) cells with TRIM21 knockdown. (G and H) A549 (G) and BEAS-2B (H) cells were treated with CHX (20 μg/mL) for the indicated times. The protein stability of SUCLG2 was detected by western blotting. (I)A549 cells were transfected with the indicated plasmids. Co-IP and western blotting were used to detect the K48-ubiquitination of SUCLG2. (J and K) Co-IP was performed in A549 cells transfected with SUCLG2 and TRIM21-siRNAs, the ubiquitination (J) and K63-lingage ubiquitination (K) of the SUCLG2 protein were detected followed by western blotting. (L) The His-SUCLG2 and HA-TRIM21 plasmids were co-transfected into A549 cells, and the cells were treated with MG132 (20 μM) for 12 h. The ubiquitination of the SUCLG2 protein was detected by co-IP and western blotting. (M) The His-SUCLG2 and HA-TRIM21 plasmids were co-transfected into A549 cells, and the cells were treated with CQ (20 μM) for 12 h. The ubiquitination of the SUCLG2 protein was detected by co-IP and western blotting.

S6

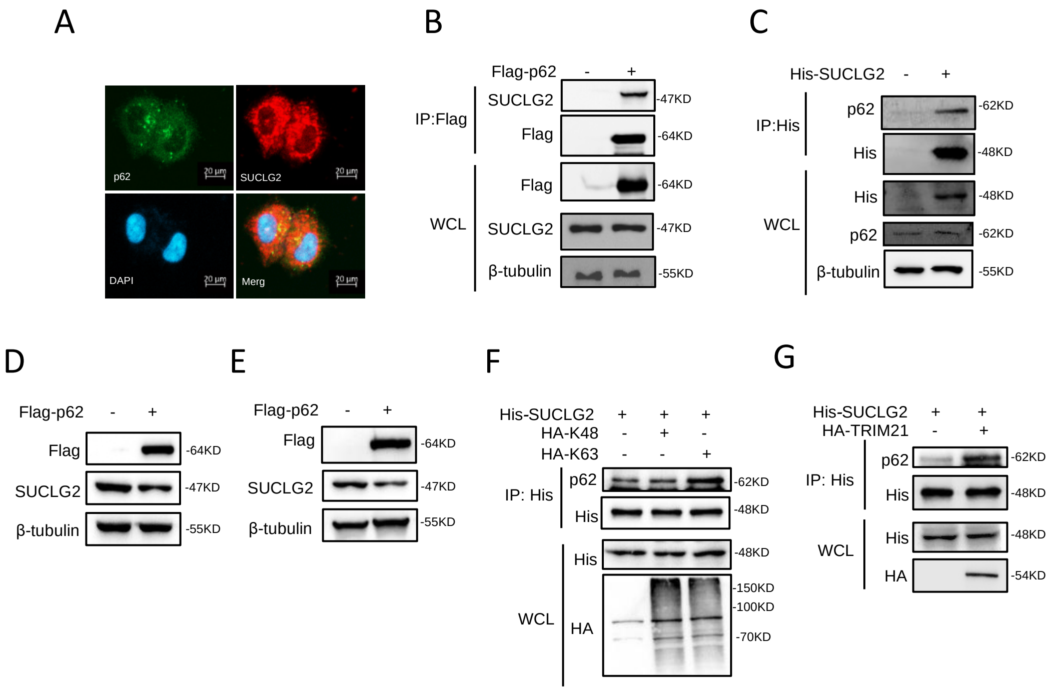

Figure S6. p62 plays an important role in the protein degradation of SUCLG2. (A) The co-localization of SUCLG2 and p62 in A549 cells was detected by immunofluorescence. (B and C) A549 cells were transfected with the pCMV-Flag-p62 (B) or pcDNA-His-SUCLG2 plasmid (C), and the interaction between SUCLG2 and p62 was detected using co-IP and western blotting. (D and E) Flag-p62 was transfected into A549 (D) and H1299 (E) cells for 48 h, and the protein level of SUCLG2 was detected by western blotting. (F) A549 cells were transfected with the indicated plasmids, followed by co-IP and western blotting. (G) Co-IP was performed in A549 cells transfected with SUCLG2 and TRIM21 before subjecting them to western blotting with an anti-p62 antibody.

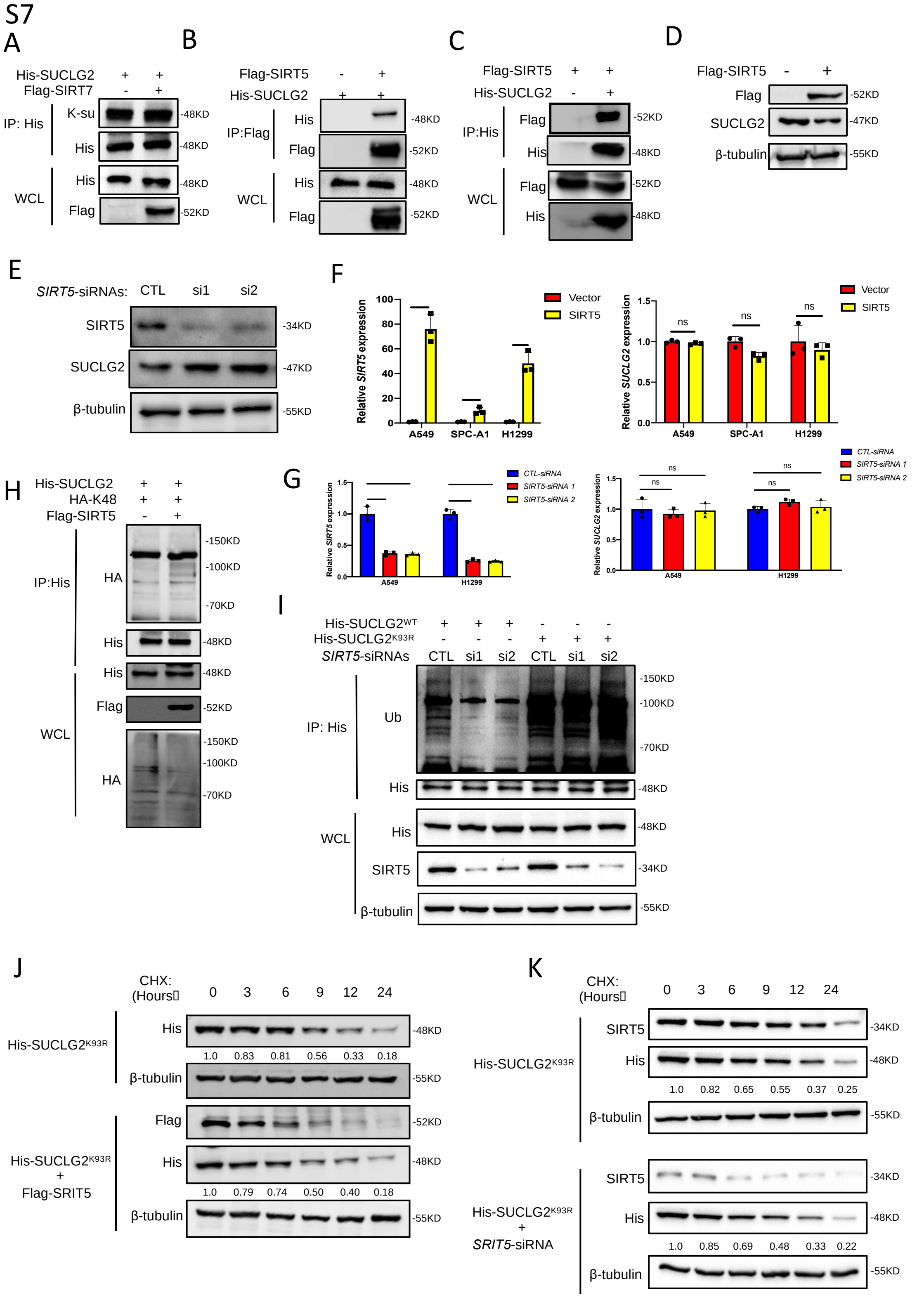

Figure S7. SIRT5 is the desuccinylase for SUCLG2. (A) Flag-SIRT7 and His-SUCLG2 were transfected into A549 cells, and the succinylation of SUCLG2 was detected by western blotting. (B and C) Flag-SIRT5 and His-SUCLG2 were

overexpressed in A549 cells, and the interaction between SIRT5 and SUCLG2 was demonstrated using co-IP and western blotting. (D and E) In H1299 cells, SIRT5 was overexpressed (D) or knocked down (E) and the SUCLG2 protein was detected by western blotting. (F and G) In A549 and H1299 cells, SIRT5 was overexpressed (F) or knocked down (G) and the mRNA expression of SUCLG2 was detected by RT-PCR. The data represent the average of three independent experiments (mean ± SD). ns, $P > 0.05$; ***$P < 0.001$. (H) The indicated plasmids were transfected into A549 cells. Co-IP and western blotting were used to detect the K48-ubiquitination of SUCLG2. (I) A549 cells were transfected with the indicated plasmid and SIRT5 siRNAs, and co-IP and western blotting were used to detect the ubiquitination level of SUCLG2. (J and K) SIRT5 was overexpressed (J) or knocked down (K) in A549 cells transfected with the His-SUCLG2^{K93R} plasmid, and the cells were treated with CHX (20 μg/mL) for 0h, 3h, 6h, 9h, 12h, and 24 h. The degradation rate of the SUCLG2 protein was detected by western blotting.
